# Machine-assisted adaptive feedback consensus method for large-scale group decision making based on reinforcement learning

**DOI:** 10.3389/fdata.2026.1921037

**Published:** 2026-09-24

**Authors:** Hongyu Yu, Xuanhua Xu, Weiwei Zhang

**Affiliations:** 1 School of Advanced Interdisciplinary Studies, Hunan University of Technology and Business, Changsha, China; 2 School of Business, Central South University, Changsha, China

**Keywords:** adaptive feedback mechanism, consensus reaching, human-machine collaboration, large-scale group decision-making, reinforcement learning

## Abstract

**Introduction:**

Traditional consensus-reaching processes in large-scale group decision making (LSGDM) rely heavily on empirically determined feedback parameters and have difficulty balancing consensus efficiency with the preservation of expert opinions. To address this problem, this paper proposes a reinforcement learning-based machine-assisted adaptive feedback consensus method.

**Methods:**

First, a hesitation degree is introduced to improve the score function of hesitant fuzzy linguistic term sets (HFLTSs), thereby reducing information loss during linguistic information transformation. Second, a comprehensive relationship matrix integrating preference similarity and social trust relationships is constructed, and K-Means clustering is employed to divide large-scale expert groups. On this basis, the consensus-reaching process is modeled as a Markov decision process (MDP), and the LSGDM consensus-reaching process is modeled as a dynamic sequential decision problem. A deep deterministic policy gradient (DDPG) agent is utilized to learn feedback adjustment strategies, enabling feedback parameters to be dynamically adjusted according to the evolution state of group opinions. Meanwhile, network text data and the TF-IDF method are combined to determine attribute weights and improve decision objectivity. Finally, an emergency decision-making case of the “Beijing-Tianjin-Hebei rainstorm” is conducted to verify the proposed method.

**Results:**

The results show that the proposed method can achieve the preset consensus threshold within fewer discussion rounds while effectively balancing the group consensus level and expert opinion retention.

**Discussion/Conclusion:**

These findings verify the effectiveness and feasibility of the proposed method, indicating that it can provide effective decision support for adaptive feedback consensus reaching in LSGDM emergency decision-making.

## 1 Introduction

In recent years, the frequent occurrence of sudden public emergencies, such as typhoons and extreme rainfall events, has posed severe challenges to social property and human safety. In such highly complex and rapidly changing scenarios, any hesitation or misjudgment during the decision-making process may lead to serious consequences. Therefore, coordinating multiple stakeholders, integrating collective intelligence, and implementing scientific and accurate emergency decision-making are of great importance (Xu and Zhou, 2024; Yin et al., 2025). In particular, the Chinese government has repeatedly emphasized the principles of “people-oriented development” and “prioritizing people's lives and safety,” while continuously improving emergency warning and response mechanisms, which places higher requirements on modern emergency management systems.

With the increasing complexity of real-world decision-making environments, Multiple Attribute Decision Making (MADM) methods have been widely applied in various fields, including risk management, resource allocation, investment evaluation, and sustainable development. In complex decision scenarios, decision makers are usually required to consider multiple evaluation criteria, uncertain information, and diverse stakeholder preferences. Therefore, effectively integrating multi-source information and supporting scientific decision-making have become important research issues. For example, in the field of natural disaster risk management, fuzzy multi-criteria decision-making approaches can effectively handle uncertainty in risk assessment and provide decision support for disaster prevention and emergency resource allocation (Alam et al., 2024). Moreover, in sustainable investment and emerging technology evaluation, the combination of artificial intelligence and fuzzy decision-making methods can comprehensively consider economic, technological, and environmental factors, thereby improving the analysis capability of complex decision problems (Yüksel et al., 2025).

However, as the scale of decision-making tasks continues to expand, traditional small-group decision-making approaches that rely on a limited number of decision makers are no longer sufficient to fully integrate complex and multi-source information. Particularly in major emergency response scenarios, the number of participating experts is usually large, and they often come from different professional backgrounds, resulting in significant differences in knowledge structures, risk perceptions, and value preferences (Xu et al., 2021; Xu and Zhu, 2023). Therefore, Large-Scale Group Decision Making (LSGDM), which can integrate diverse opinions and improve decision-making robustness, has gradually become an important approach for addressing complex emergency decision problems. Nevertheless, how to coordinate conflicts among experts and achieve effective consensus while ensuring decision-making efficiency remains a critical challenge in both theoretical research and practical applications.

In the process of large-scale group decision-making (LSGDM), when a consensus has not been reached among the group members, the opinions of the subgroups obtained through clustering are iteratively evolved until the consensus threshold is satisfied. This iterative procedure is referred to as the Consensus Reaching Process (CRP; Wan et al., 2020; Herrera et al., 2001). As the core component of LSGDM, the efficiency and quality of CRP directly determine the success or failure of emergency responses. A review of the existing literature reveals that early approaches—such as the two-stage consensus method (Gao et al., 2022; Zhang et al., 2023; Li et al., 2021), the minimum-cost consensus method (Lu et al., 2024; Ji et al., 2022; Zhang et al., 2018), and trust-based consensus methods (Zhang et al., 2021; Yu et al., 2021; Zhang et al., 2022)—have established a relatively comprehensive theoretical foundation. With the growing recognition of the complexity inherent in expert behaviors, research perspectives have progressively shifted from static rule-based mechanisms toward dynamic adaptive frameworks. For instance, Shang et al. (2023) employed similarity-driven Louvain dynamic clustering in an intuitionistic fuzzy environment to capture the structural evolution of subgroups induced by experts' compromise behaviors, and designed a two-stage switching strategy characterized by “low-consensus interaction and high-consensus automation” to balance decision efficiency and acceptability, while simultaneously incorporating intra-subgroup negotiation and an “exit-delegation” mechanism to manage non-cooperative behaviors. Concurrently, artificial intelligence techniques have begun to demonstrate promising potential in the field of decision-making. Wang et al., drawing upon cognitive psychology, confirmed that decision-makers' prior beliefs and task-specific knowledge significantly constrain the effectiveness of human-machine collaboration (Wang et al., 2026). Tan et al. (2026) coupled trust structures with preference information and employed the Deep Q-Network (DQN) algorithm to achieve adaptive generation of consensus strategies. Hassani et al. (2023) adopted a Dual-Agent Deep Deterministic Policy Gradient (DDPG) framework to optimize feedback parameters and weight allocation in a coordinated manner. (Tu et al. 2026) combined Multi-Agent Deep Q-Network (MADQN) with social network clustering, effectively reducing the number of iterations and enhancing robustness against non-cooperative behaviors. Shen et al. (2026) further accommodated both interactive and automated decision-making modes within a reinforcement learning paradigm. In addition, Yang and Wang (2026) approached the problem from a psychological behavior perspective, optimizing subgroup weight calculation using the Hamacher T-norm operator, thereby refining the modeling of experts' self-confidence levels and psychological disparities. However, existing reinforcement learning-assisted consensus methods mainly focus on parameter optimization issues, with less consideration given to the characteristics of dynamic opinion evolution within large-scale expert groups. Therefore, this paper does not aim to improve the DDPG algorithm structure, but rather explores how to embed deep reinforcement learning into the dynamic consensus feedback process, enabling the agent to generate adaptive feedback strategies based on real-time group states.

Based on the above analysis, to address the problem that traditional consensus-reaching processes have fixed feedback parameters and struggle to dynamically adapt to changes in group states, this paper proposes a reinforcement learning-based machine-assisted adaptive feedback consensus method for large-scale group decision making. The main contributions of this paper include: to tackle the issue that traditional HFLTS score functions are prone to misjudging equal values, hesitation degree information is introduced to improve the score function, thereby enhancing the expressiveness of uncertain linguistic information; a comprehensive relationship matrix is constructed by integrating expert preference similarity and social trust relationships, and K-Means clustering is employed to partition the large-scale expert group; the consensus-reaching process is modeled as a Markov decision process, and a DDPG agent is utilized to learn feedback adjustment strategies, enabling feedback parameters to be dynamically adjusted according to the evolution of group opinions, thus achieving adaptive optimization of the feedback regulation process; finally, attribute weights are obtained by combining network text data and the TF-IDF method, and a case study is conducted to verify the effectiveness of the proposed method. Motivated by the above analysis, this paper proposes an adaptive feedback consensus reaching method for human-machine collaborative LSGDM based on reinforcement learning, to address the issue that fixed feedback parameters in traditional CRP are incapable of dynamically adapting to the evolution of the group state. The main contributions of this paper are fourfold: (1) to ameliorate the problem that conventional HFLTS score functions are prone to misjudgment due to equivalent values, hesitation degree information is incorporated to refine the score function, thereby enhancing the expressive capacity for uncertain linguistic information; (2) a comprehensive relationship matrix is constructed by integrating expert preference similarity with social trust relations, upon which K-Means clustering is performed to realize the partition of large-scale experts into subgroups; (3) the CRP is modeled as a Markov Decision Process (MDP), and the DDPG algorithm is employed to dynamically generate continuous feedback parameters, thereby achieving adaptive optimization of the feedback adjustment process; and (4) attribute weights are derived from online textual data using the TF-IDF approach, and a case study is conducted to validate the effectiveness of the proposed method.

## 2 Materials and methods

### 2.1 Problem formulation

Consider a Large-Scale Group Decision-Making (LSGDM) problem, in which there exists a set of alternatives and a set of evaluation attributes *C* = {*c*_*j*_|*j* = 1, 2, …, *N*}, with the corresponding attribute weight vector denoted as *w* = {*w*_*j*_|*j* = 1, 2, …, *N*}. The decision-making large group consists of *M* experts, denoted as Ω = {*e*_*i*_|*i* = 1, 2, …, *M*; *M*≥20}. Each expert *e*_*i*_ provides an initial preference matrix in the form of Hesitant Fuzzy Linguistic Term Sets (HFLTS), denoted as $H^{i}={\left\{H_{lj}^{i}\right\}}_{P\times N}$. The objective of this paper is to adaptively drive the feedback consensus reaching process through deep reinforcement learning, incorporate public social information to optimize the attribute weights, and ultimately output the optimal alternative.

### 2.2 Refined hesitant fuzzy linguistic score function

Linguistic terms have been widely employed in decision-making problems. However, as decision-making problems have become increasingly complex and dynamic, decision-makers tend to express their preferences using multiple linguistic terms or more sophisticated linguistic expressions. To handle such hesitant situations, Rodriguez et al. (2011) introduced the concept of Hesitant Fuzzy Linguistic Term Sets (HFLTS).

**Definition 1** (Rodriguez et al., 2011): Let *S* = {*s*_0_, *s*_1_, …, *s*_τ_} be a linguistic term set (LTS) with granularity (τ+1), and let *H*_*S*_ be a hesitant fuzzy linguistic term set (HFLTS), which is an ordered finite subset of consecutive linguistic terms of *S*:


$$\begin{array}{l} H_{S}=\left\{s_{\delta_{l}}|l=1,2,\ldots ,\#H_{S};s_{\delta_{l}}\in S\right\} \end{array}$$

where *#H*_*S*_ is the number of linguistic terms in *H*_*S*_.

Owing to the heterogeneity of decision-makers' knowledge backgrounds, decision-making perspectives, and personal preferences, their judgments on the same issue may differ considerably. Consequently, multi-granularity linguistic terms are commonly adopted for preference expression to ensure the relative accuracy of preference representation. For example, a linguistic term set with granularity 5, *S*^5^ = {very poor, poor, fair, good, very good}, and a set with granularity 3, *S*^3^ = {low, medium, high}.

**Definition 2** (Xu et al., 2023): For an *H*_*S*_ = {*s*_δ_*l*__|*l* = 1, 2, …, *#H*_*S*_}, the score function *F*(*H*_*S*_) is defined as follows:


$$\begin{array}{l} F\left(H_{S}\right)=\bar{\delta }-{\frac{\frac{1}{\#H_{S}}\sum_{l=1}^{\#H_{S}}\left(\delta_{l}-\bar{\delta }\right)}{var\left(\tau \right)}}^{2} \end{array}$$ (1)

where $\bar{\delta }=\frac{1}{\#H_{S}}\sum_{l=1}^{\#H_{S}}\delta_{l}$, and $var\left(\tau \right)=\frac{{\left(0-\tau /2\right)}^{2}+\ldots +{\left(\tau -\tau /2\right)}^{2}}{\tau +1}$. The following decision rules apply: If $F\left(H_{S}^{1}\right)>F\left(H_{S}^{2}\right)$, then $H_{S}^{1}>H_{S}^{2}$;If $F\left(H_{S}^{1}\right)=F\left(H_{S}^{2}\right)$, then $H_{S}^{1}=H_{S}^{2}$.

To address the issue that traditional score functions are prone to misjudgment due to equivalent values under varying decision-making uncertainties—for instance, when a multi-element set and a single-element set yield identical scores—this paper introduces the hesitation degree *HD*(*H*_*S*_) to characterize the extent of experts' hesitation when articulating linguistic evaluations.

**Definition 3** (Zhong et al., 2022): For an HFLTS *H*_*S*_ = {*s*_δ_*l*__|*l* = 1, 2, …, *#H*_*S*_}, the hesitation degree is defined as follows:


$$\begin{array}{l} HD\left(H_{S}\right)=\sqrt{\frac{\sum_{l=1}^{\#H_{S}}{\left(\gamma^{l}-\bar{\gamma }\right)}^{2}}{\#H_{S}}} \end{array}$$ (2)

where $\gamma^{l}=\frac{l}{\tau }$ denotes the membership degree of the linguistic term in *H*_*S*_, τ is the maximum subscript of the adopted linguistic term set (LTS), $\bar{\gamma }=\frac{\sum_{l=1}^{\#H_{S}}\gamma^{l}}{\#H_{S}}$ is the mean of the membership degrees of the linguistic terms in *H*_*S*_, and *#H*_*S*_ is the number of linguistic terms in *H*_*S*_.

In the environment of hesitant fuzzy linguistic term sets (HFLTS), experts may simultaneously provide multiple linguistic terms to express their degree of hesitation. This paper introduces a hesitation degree indicator to quantify the uncertainty embedded in linguistic evaluation sets. A higher value of this indicator indicates greater uncertainty in expert opinions, and consequently, a lower reliability of the evaluations. Compared with merely relying on linguistic evaluation values, incorporating hesitation information can more comprehensively capture the psychological uncertainty in experts' expression processes, thus being particularly suitable for large-scale group decision-making scenarios characterized by high complexity and uncertainty.

The selection of the hesitation degree indicator in this paper is mainly based on the following two considerations:

(1) It aligns with the essential characteristics of hesitant fuzzy linguistic expressions. In large-scale group decision making, due to differences in experts' knowledge backgrounds, risk preferences, and access to information, they often find it difficult to provide a single deterministic evaluation and tend to offer multiple possible linguistic values. The hesitation degree can effectively reflect this trait.
(2) It serves to depict the reliability of expert opinions. Traditional HFLTS methods primarily focus on the transformation of linguistic information but neglect the inherent uncertainty within the evaluations. Introducing the hesitation degree indicator can simultaneously reflect the degree of certainty, information reliability, and preference stability in expert opinions, thereby providing an important basis for further improving the rationality of expert weight calculation.

Accordingly, the refined score function is constructed as follows:


$$\begin{array}{l} \rho \left(H_{S}\right)=\left(1-HD\left(H_{S}\right)\right)\times F\left(H_{S}\right) \end{array}$$ (3)

For expert *e*_*i*_, the preference matrix is denoted as $H^{i}={\left\{H_{lj}^{i}\right\}}_{P\times N}$, where each element $H_{lj}^{i}$ represents the evaluation value of expert *e*_*i*_ in the form of a hesitant fuzzy linguistic term set with respect to alternative *x*_*l*_ and attribute *c*_*j*_. First, Equation 3 is applied to obtain $\rho \left(H_{lj}^{i}\right)$. Then, Equation 4 is employed to normalize the resulting values $v_{lj}^{i}$ into the interval [0, 1], yielding the final numerical preference matrix $V^{i}={\left\{v_{lj}^{i}\right\}}_{P\times N}$ for expert *e*_*i*_:


$$\begin{array}{l} v_{lj}^{i}=\frac{\rho \left(H_{lj}^{i}\right)}{\tau } \end{array}$$ (4)

It should be noted that the constant 6 used in the above normalization process originates from the seven-granularity linguistic evaluation set defined in this paper as *S* = {*s*_0_, *s*_1_, *s*_2_, *s*_3_, *s*_4_, *s*_5_, *s*_6_}, where the maximum subscript of the linguistic terms is 6. This parameter is not an empirically determined threshold, but is determined by the scale of the linguistic evaluation system. When the granularity of the linguistic term set changes, the normalization constant should be adjusted accordingly to the maximum subscript of the linguistic set. For example, if a five-granularity linguistic term set *S* = {*s*_0_, *s*_1_, *s*_2_, *s*_3_, *s*_4_} is adopted, the normalization constant should be adjusted to 4. This design ensures the generality and consistency of the score function across different linguistic evaluation systems.

**Definition 4**: Inspired by the concept of Hamming distance (Zhang et al., 2017), for experts *e*_*i*_ and *e*_*h*_ with preference matrices $H^{i}={\left\{H_{lj}^{i}\right\}}_{P\times N}$ and $H^{h}={\left\{H_{lj}^{h}\right\}}_{P\times N}$, respectively, the distance between $H_{lj}^{i}$ and $H_{lj}^{h}$ is computed by first converting them into numerical preferences $v_{lj}^{i}$ and $v_{lj}^{h}$ using Equation 4, and then calculating the distance as follows:


$$\begin{array}{l} d\left(H_{lj}^{i},H_{lj}^{h}\right)=|v_{lj}^{i}-v_{lj}^{h}| \end{array}$$ (5)

### 2.3 Social network analysis

In the field of decision-making, decision experts are not isolated individuals and are inevitably influenced by others. Therefore, under complex and dynamic decision-making environments, it is necessary to conduct Social Network Analysis (SNA) of experts.

Social networks can be represented in three forms: network graphs, adjacency matrices, and algebraic expressions (Xu and Zhang, 2020), as illustrated below:

**Network graph**: The relationship between two experts can be represented by an edge connecting the corresponding vertices in the graph.

**Social relation matrix**: Each element *ST*_*hi*_ in the social relation matrix *Tr* = [*S*_*T*_*hi*_]*M*×*M*_ represents the trust value assigned by expert *e*_*h*_ to expert *e*_*i*_.

**Algebraic expression**: An expression such as *e*_*h*_*Re*_*i*_ can denote the trust relationship between decision-makers.

**Definition 5** (Xu and Zhang, 2020): Let the normalized in-degree and normalized out-degree of expert *e*_*i*_ in the social network be denoted as *IND*_*i*_ and *OUTD*_*i*_, respectively, which are computed as follows:


$$\begin{array}{l} IND_{i}=\frac{IN_{i}}{L} \end{array}$$ (6)

$$\begin{array}{l} OUTD_{i}=\frac{OUT_{i}}{L} \end{array}$$ (7)

where *IN*_*i*_ and *OUT*_*i*_ denote the number of directed edges with expert *e*_*i*_ as the destination and source, respectively, and *L* is the total number of edges in the social network graph.

## 3 Methodology fundamentals

### 3.1 Expert clustering by integrating preferences and trust relations

Many existing studies cluster large-scale experts based solely on either preference information or trust relations. However, a scenario may arise in which two experts share a high level of mutual trust yet hold divergent preferences. To mitigate the clustering distortion caused by such preference divergence in the presence of high trust, this paper fuses preference similarity and trust values to construct a comprehensive relationship.

**Definition 6**: For experts *e*_*i*_ and *e*_*h*_, their preference expressions in the form of HFLTS with respect to alternative *x*_*l*_ and attribute *c*_*j*_ are denoted as$H_{lj}^{h}$ and $H_{lj}^{i}$, respectively. The similarity between them with respect to alternative *x*_*l*_ and attribute *c*_*j*_ is computed as follows:


$$\begin{array}{l} sm_{hi}^{lj}=1-d\left(H_{lj}^{i},H_{lj}^{h}\right)=1-|v_{lj}^{i}-v_{lj}^{h}| \end{array}$$ (8)

The overall opinion similarity between experts *e*_*i*_ and *e*_*h*_ is computed as follows:


$$\begin{array}{l} SD_{hi}=\frac{1}{P\times N}\overset{P}{\underset{l=1}{\sum }}\overset{N}{\underset{j=1}{\sum }}\left(1-|v_{lj}^{i}-v_{lj}^{h}|\right) \end{array}$$ (9)

The preference similarity and trust value between experts *e*_*h*_ and *e*_*i*_ are denoted as *SD*_*hi*_ and *ST*_*hj*_, respectively. The former can be obtained using Equation 9, while the latter is derived from the trust relation matrix. On this basis, the new comprehensive relationship between experts *e*_*i*_ and *e*_*h*_ is computed as follows:


$$\begin{array}{l} SN_{hi}=\alpha \times ST_{hi}+\left(1-\alpha \right)\times SD_{hi} \end{array}$$ (10)

where α ∈ [0, 1] is a balance coefficient [set to α = 0.5 in this paper following (Lu et al., 2024)]. Based on the SN matrix, the K-Means clustering method (Lu et al., 2021) is employed to partition the large-scale experts into *K* clusters, denoted as Ω^*^ = {*C*^1^, *C*^2^, …, *C*^*k*^}, where the number of experts in cluster *C*^*K*^ is denoted as *n*_*k*_.

In large-scale group decision making, clustering based solely on preference similarity may lead to the neglect of long-term trust relationships among experts, failing to reflect social influence factors; while relying solely on social trust relationships may overlook the actual differences in expert opinions. Therefore, this paper integrates preference similarity and social trust relationships, enabling the simultaneous characterization of both the proximity of expert opinions and the degree of influence among expert relationships. Regarding the choice of the fusion coefficient θ, a sensitivity analysis is conducted in this paper (see Section 5.2 for details), testing the clustering performance under different values of θ = 0.3, 0.5, 0.7. The results show that when θ = 0.5, the two types of information are assigned equal importance, yielding the highest silhouette coefficient and the best clustering performance. Therefore, this paper adopts θ = 0.5 as the default value. In addition, the K-Means clustering method is employed, and the optimal number of subgroups is determined by the silhouette coefficient to ensure the rationality of the clustering results.

### 3.2 Determination of cluster and expert weights

#### 3.2.1 Determination of cluster weights

We introduce the Hamacher t-norm operator (Urena et al., 2019), defined as $\left(a,b\right)=\frac{a+b-2ab}{1-ab}$, to fuse the scale weight λ_*k*_ and the credibility weight *Tλ*_*k*_ for computing the normalized comprehensive weight *u*_*k*_ of cluster *C*^*K*^:


$$\begin{array}{l} {\text{λ}}_{k}=\frac{n_{k}}{M},T{\text{λ}}_{k}=\frac{\sum_{i=1,e_{i}\in C^{k}}^{n_{k}}TD_{i}}{\sum_{k=1}^{K}\sum_{i=1,e_{i}\in C^{k}}^{n_{k}}TD_{i}} \end{array}$$ (11)

$$\begin{array}{l} u_{k}=\frac{\varphi \left({\text{λ}}_{k},T{\text{λ}}_{k}\right)}{\sum_{k=1}^{K}\varphi \left({\text{λ}}_{k},T{\text{λ}}_{k}\right)} \end{array}$$ (12)

#### 3.2.2 Determination of expert weights

In a social network, experts who interact more frequently or trust each other more deeply are assigned greater weights. The former can be measured by the normalized in-degree and normalized out-degree, while the latter can be quantified by the credibility degree. By integrating the network topology degree and the credibility degree, the topological weight ${\tilde{\omega }}_{i}$ and the final normalized weight ω_*i*_ of expert *e*_*i*_ are computed as follows:


$$\begin{array}{l} {\tilde{\omega }}_{i}=\frac{IND_{i}+OUTD_{i}}{\sum_{i=1}^{M}\left(IND_{i}+OUTD_{i}\right)} \end{array}$$ (13)

$$\begin{array}{l} \omega_{i}=\frac{\varphi \left(\omega_{i},TD_{i}\right)}{\sum_{i=1}^{M}\varphi \left(\omega_{i},TD_{i}\right)} \end{array}$$ (14)

### 3.3 Consensus measurement

Consensus reaching plays a pivotal role in large-scale group decision-making. Once the group consensus level attains the consensus threshold γ, the process can proceed to the alternative selection stage. Otherwise, feedback adjustment is required until the consensus threshold is satisfied, after which the alternative selection process is conducted.

#### 3.3.1 Consensus calculation

Let the decision matrix of cluster *C*^*k*^ be denoted as $R^{k}={\left\{c_{lj}^{k}\right\}}_{P\times N}$, Since different subgroups contain varying numbers of experts and different sums of global weights after clustering, directly using the global expert weights to compute subgroup preferences may lead to underestimation of the contributions of experts within certain subgroups. Therefore, this paper first performs a re-normalization of the expert weights within each subgroup. The local weight of expert *e*_*i*_ in subgroup *C*^*k*^ is defined as follows:


$$\begin{array}{l} {\bar{\omega }}_{i}^{k}=\frac{\omega_{i}}{\sum_{e_{j}\in C^{k}}\omega_{j}} \end{array}$$ (15a)

where ω_*i*_ denotes the global weight of expert *e*_*i*_, and ${\bar{\omega }}_{i}^{k}$ denotes the re-normalized local weight of expert *e*_*i*_ within subgroup *C*^*k*^, satisfying the following condition:


$$\begin{array}{l} \underset{e_{i}\in C^{k}}{\sum }{\bar{\omega }}_{i}^{k}=1 \end{array}$$

Based on the above local weights, the elements of the comprehensive preference matrix for the *k*-th subgroup are computed as follows:


$$\begin{array}{l} c_{lj}^{k}=\underset{e_{i}\in C^{k}}{\sum }{\bar{\omega }}_{i}^{k}v_{lj}^{i}=\underset{e_{i}\in C^{k}}{\sum }\frac{\omega_{i}}{\sum_{e_{j}\in C^{k}}\omega_{j}}v_{lj}^{i} \end{array}$$ (15)

where $c_{lj}^{k}$ represents the comprehensive preference value of the *k*-th subgroup for alternative *l* under attribute *j*; $v_{lj}^{i}$ denotes the preference value of expert *e*_*i*_ for alternative *l* with respect to attribute *j*; ω_*i*_ is the global weight of expert *e*_*i*_; *C*^*k*^ denotes the *k*-th expert subgroup; and ${\bar{\omega }}_{i}^{k}$ is the local weight of expert *e*_*i*_ within subgroup *C*^*k*^.

After obtaining the comprehensive preference matrices of each subgroup, the subgroup preferences are further aggregated according to the subgroup weights to obtain the comprehensive decision matrix *G* = {_*g*_*lj*_}*P*×*N*_ of the entire expert group, where *g*_*lj*_ is computed as follows:


$$\begin{array}{l} g_{lj}=\overset{K}{\underset{k=1}{\sum }}u_{k}\cdot c_{lj}^{k} \end{array}$$ (16)

For the (*l, j*)-th element, the element-level consensus level of expert *e*_*i*_ is computed as follows:


$$\begin{array}{l} c\varepsilon_{lj}^{i}=1-|v_{lj}^{i}-g_{lj}| \end{array}$$ (17)

For a given alternative *x*_*l*_, the consensus level of expert *e*_*i*_ is computed as follows:


$$\begin{array}{l} c\varepsilon_{l}^{i}=\frac{1}{N}\overset{N}{\underset{j=1}{\sum }}c\varepsilon_{lj}^{i} \end{array}$$ (18)

The consensus level of expert *e*_*i*_ is computed as follows:


$$\begin{array}{l} CL^{i}=\frac{1}{p}\overset{p}{\underset{l=1}{\sum }}c\varepsilon_{l}^{i} \end{array}$$ (19)

The consensus level of cluster *C*^*k*^ is computed as follows:


$$\begin{array}{l} CLC^{k}=\overset{n_{k}}{\underset{i=1,e_{i}\in C^{k}}{\sum }}\omega_{i}\cdot CL^{i} \end{array}$$ (20)

The group consensus level is computed as follows:


$$\begin{array}{l} GCL=\overset{K}{\underset{k=1}{\sum }}u_{k}\cdot CLC^{k} \end{array}$$ (21)

#### 3.3.2 Feedback adjustment

Once the consensus measurement is completed, the recommendation mechanism is activated to generate suggestions for the experts identified according to the following identification and direction rules.

##### 3.3.2.1 Identification rules

Identify the clusters that do not satisfy the consensus threshold, and select all experts in the cluster with the minimum consensus level:


$$\begin{array}{l} C=\left\{k|CLC^{k}<\gamma ;k=1,2,\ldots ,K\right\}\\ C_{\min}=\left\{k|\min\left(CLC^{k}\right);k\in C\right\}\\ EXPCH=\left\{\left(k,i\right)|e_{i}\in C^{k}\cap k\in C\min\right\} \end{array}$$ (22)

Identify the set of alternatives to be adjusted, denoted as *ALT*:


$$\begin{array}{l} ALT=\left\{\left(k,i,l\right)|\left(k,i\right)\in EXPCH\cap c\varepsilon_{l}^{i}<\gamma \right\} \end{array}$$ (23)

Identify the set of opinions to be adjusted, denoted as *APS*:


$$\begin{array}{l} APS=\left\{\left(k,i,l,j\right)|\left(k,i,l\right)\in ALT\cap c\varepsilon_{lj}^{i}<\gamma \right\} \end{array}$$ (24)

##### 3.3.2.2 Adjustment rules

The personalized adjustment suggestions are generated as follows:


$$\begin{array}{l} v_{lj}^{i}\left(t+1\right)=\left(1-\delta \right)v_{lj}^{i}\left(t\right)+\delta g_{lj}\left(t\right) \end{array}$$ (25)

The opinion retention degree *RD*^*i*^ of expert *e*_*i*_ is computed as follows:


$$\begin{array}{l} RD^{i}=1-\frac{1}{P\times N}\overset{P}{\underset{l=1}{\sum }}\overset{N}{\underset{j=1}{\sum }}\left(\left|v_{lj}^{i}\left(t\right)-v_{lj}^{i}\left(t+1\right)\right|\right) \end{array}$$ (26)

where $v_{lj}^{i}\left(t+1\right)$ denotes the adjusted evaluation value of expert *e*_*i*_, $v_{lj}^{i}\left(t\right)$ denotes the evaluation value of expert *e*_*i*_ at round *t* with respect to alternative *x*_*l*_ and attribute *c*_*j*_,*g*_*lj*_(*t*) denotes the group evaluation value at round *t* with respect to alternative *x*_*l*_ and attribute *c*_*j*_, and δ ∈ [0, 1] is the feedback parameter.

Regarding the adjustment rules, the magnitude of the feedback parameter δ significantly influences the CRP. In many existing studies, this value is determined based on experts' historical experience. In contrast, this paper employs a reinforcement learning algorithm to determine the feedback parameter δ, as detailed in Section 3.4.

### 3.4 Adaptive adjustment method for feedback parameters based on reinforcement learning

In the process of large-scale group consensus reaching, traditional fixed or linearly adjusted parameters often face a dilemma: excessively large step sizes may lead to over-adjustment of expert preferences, while excessively small step sizes may result in a significant increase in the number of discussion rounds. In view of this, this paper models the consensus reaching process as a Markov decision process (MDP; Urena et al., 2019). Given that feedback adjustment is inherently a control problem within a continuous action space, the Deep Deterministic Policy Gradient (DDPG) algorithm (Hassani et al., 2023) exhibits superior architectural adaptability for continuous-domain control tasks compared to discrete policy algorithms. Therefore, this paper introduces a DDPG-driven approach that leverages the Actor-Critic architecture to solve and optimize continuous feedback control actions, aiming to generate smoother and more adaptive adjustment parameters in dynamic environments.

**State Space** **S:** The state at round *t* is defined as $s_{t}=\left(d\left(H_{1}^{t},H_{c}^{t}\right),\left(H_{2}^{t},H_{c}^{t}\right),\ldots ,d\left(H_{M}^{T},H_{c}^{t}\right)\right)$, where $d\left(H_{i}^{t},H_{c}^{t}\right)$ denotes the hesitant fuzzy linguistic distance between cluster *i* and the group center preference at round *t*. This distance vector reflects the degree of deviation of each cluster from the group center, and thus serves as a critical characterization of the environmental state. To ensure that the state satisfies the Markov property, this paper further extends the state space by defining the state variable as *s*_*t*_ = (*d*_*t*_, *C*_*t*_, *R*_*t*_, *I*_*t*_), where *d*_*t*_ is the vector of opinion deviation degrees of experts, *C*_*t*_ is the current group consensus level, *R*_*t*_ is the historical feedback adjustment effect (i.e., the feedback parameters in the previous round and the corresponding consensus improvement), and *I*_*t*_ is the opinion change trend (i.e., statistics of the rate of opinion change between two adjacent rounds). The extended state space can more comprehensively describe the dynamic characteristics of the current consensus environment. Given the current state *s*_*t*_, the next state *s*_(*t*+1)_ depends only on the current state and the action *a*_*t*_ taken by the agent, thus satisfying the Markov property. This is because the state variables already contain the main information required for future feedback decisions: the current opinion distribution (*d*_*t*_), the consensus level (*C*_*t*_), and the historical feedback effect (*R*_*t*_). These sufficient statistics cover the key dynamic information during the consensus evolution process.

**Action Space** **A:** The action is defined as *a*_*t*_ = δ_*t*_ ∈ [0, 1], which determines the magnitude of opinion adjustment for experts at the current round.

**Temporal Bi-Objective Piecewise Reward Function:**

The reward function comprehensively considers the improvement of group consensus, the reduction of discussion rounds, and the preservation of expert opinion retention, aiming to balance consensus efficiency and individual satisfaction:


$$\{\begin{array}{l} \frac{\sum_{i=1}^{M}RD^{i}}{n\times \delta }\;T\leq 6\\ \frac{\sum_{i=1}^{M}RD^{i}}{M\times \delta \times T}\;T>6 \end{array}$$ (27)

where *GCL*_*t*_ and *GCL*_*t*+1_ denote the group consensus levels at rounds *t* and *t*+1, respectively, *T*denotes the number of discussion rounds, and *RD*^*i*^ denotes the opinion retention degree of expert *e*_*i*_. Under the premise of ensuring group consensus growth, a larger reward *r* is obtained by maintaining relatively small values of δ and the number of discussion rounds *T*.

In this paper, the consensus process is modeled as a Markov decision process *M* = (*S, A, R, T*, γ). The state *s*_*t*_ represents the consensus state at round *t*, including group consensus level, expert deviation degree, and inter-subgroup opinion differences; the action space *a*_*t*_ = λ_*t*_ denotes the feedback adjustment intensity; the state transition process is that after the agent executes the feedback action, the new expert opinion state is generated by the feedback adjustment rule, i.e., *s*_*t*_→*s*_(*t*+1)_. Since the current state *s*_*t*_ already contains sufficient statistics such as group consensus and expert deviation degree, the next state *s*_(*t*+1)_ depends only on the current state and action, satisfying the Markov property. The reward function adopts a multi-objective design *R*_*t*_ = ω_*c*_·*R*_*c*_+ω_*e*_·*R*_*e*_−ω_*l*_·*R*_*l*_, where *R*_*c*_ is the consensus improvement reward, *R*_*e*_ is the expert preference preservation reward, and *R*_*l*_ is the feedback round penalty, with ω_*c*_, ω_*e*_, ω_*l*_ as weight coefficients. This design ensures that the agent not only pursues rapid consensus but also avoids excessively modifying expert opinions.

**Policy Optimization and Execution:**

The δ−*Agent* is internally composed of online Actor-Critic networks and their corresponding target networks. At each interaction round, the system feeds the state *s*_*t*_ into the online Actor network π(*s*|θ^π^), which outputs an action*a*_*t*_, and the Critic network *Q*(*s, a*|θ^*Q*^) evaluates the action. The interaction tuple (*s*_*t*_, *a*_*t*_, *r*_*t*_, *s*_*t*+1_) is stored in the experience replay buffer *R*. The Critic network is updated by minimizing the Temporal Difference (TD) error:


$$\begin{array}{l} L\left(\theta^{Q}\right)=1/J{\sum_{i=1}^{J}\left(y_{i}-Q\left(s_{i},a_{i}|\theta^{Q}\right)\right)}^{2},y_{i}=r_{i}\\ \;+\gamma Q^{'}\left(s_{i+1},\pi^{'}\left((s_{i+1}|\theta^{\pi^{'}}\right)|\theta^{Q^{'}}\right) \end{array}$$ (28)

Finally, the Actor network is optimized using the policy gradient theorem, and the target network parameters are progressively updated via a soft update coefficient. The pseudo-code is presented in Algorithm 1.

Algorithm 1 Interaction between the agent and the decision-making environment. 
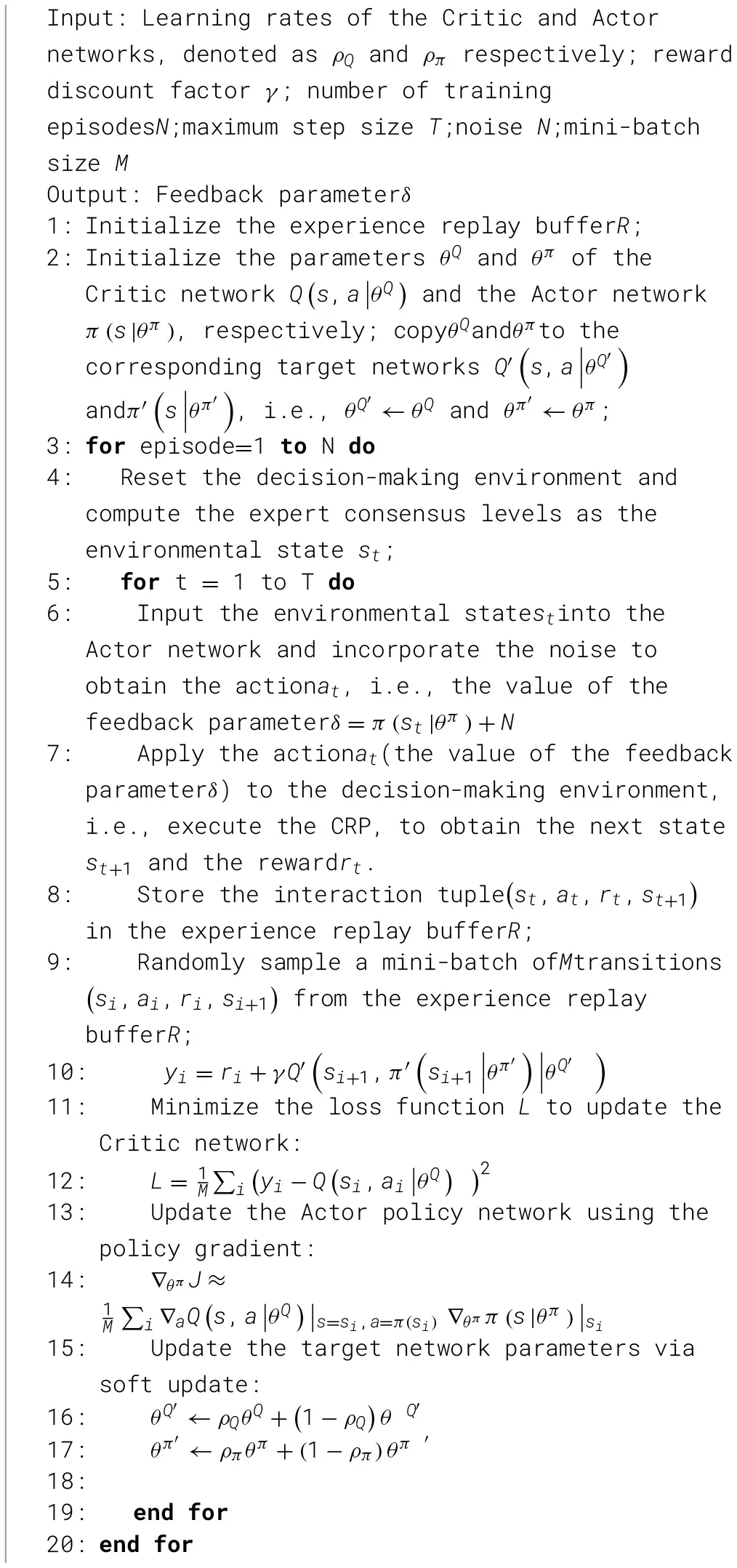

During the training phase, the parameters of the Actor and Critic networks are updated through interactions with the environment using historical consensus state samples. Specifically, the system first collects consensus evolution data from multiple large-scale group decision-making scenarios as the training set. In each round of interaction, the Actor network outputs feedback parameters based on the current state, while the Critic network evaluates the value of the action. The Critic network is updated by minimizing the temporal difference (TD) error, and the Actor network is optimized using the policy gradient theorem. After the training converges, the learned policy ππ is applied to new decision-making tasks, enabling automatic generation of feedback parameters. This training-application separation mechanism allows the model to learn sufficiently offline, and then quickly generate adaptive feedback strategies during online application. The specific process is illustrated in Figure 1.

**Figure 1:**
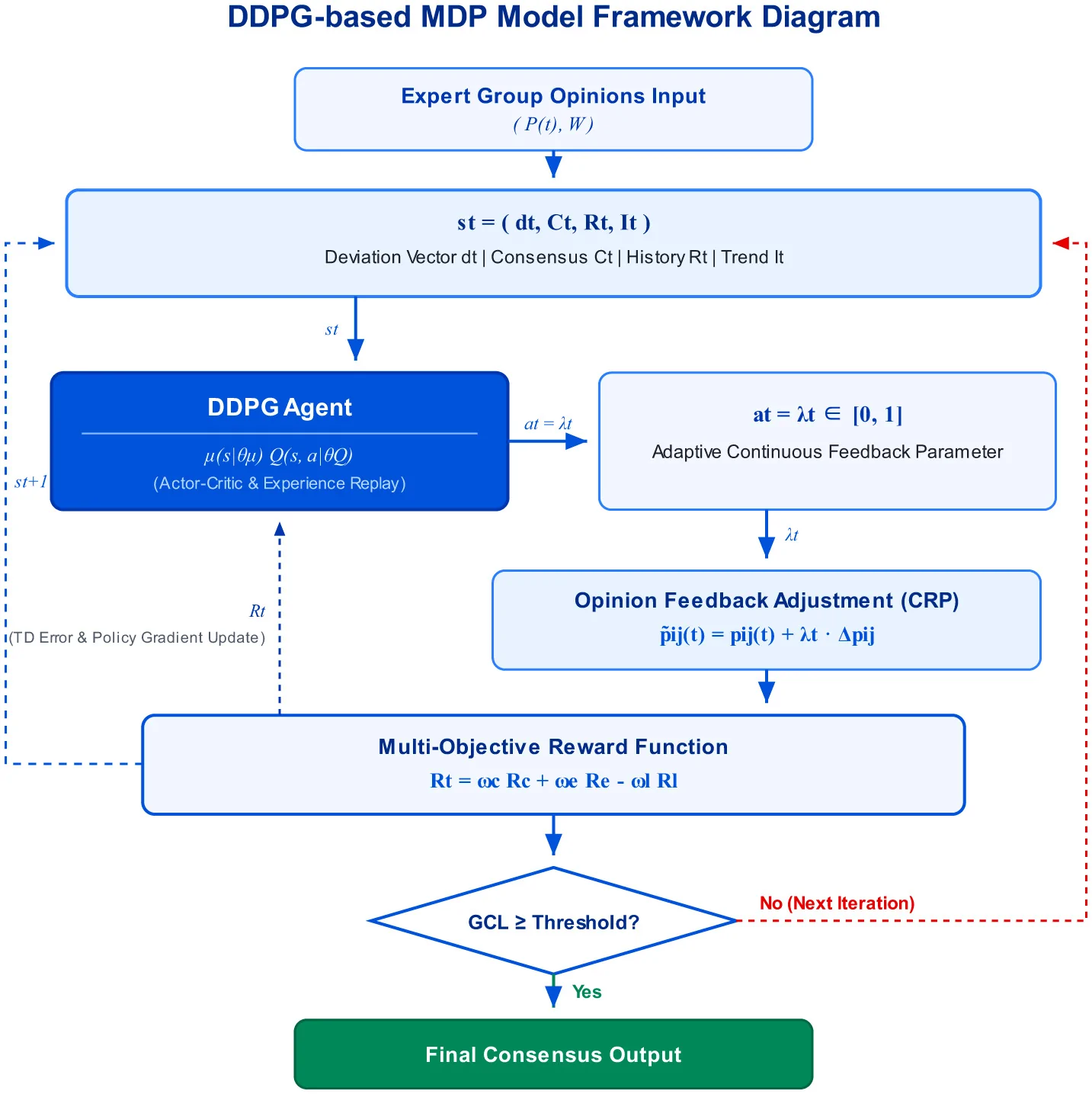
DDPG-based MDP model framework diagram.

This framework illustrates the complete decision process of the MDP model: (1) Expert group state input: the system collects the preference information of each subgroup in the current round, calculates the group consensus level, expert deviation degree, historical feedback effect, and opinion change trend, and constructs the state vector *s*_*t*_; (2) DDPG agent decision-making: the Actor network outputs a continuous feedback parameter *a*_*t*_ = λ_*t*_ based on the current state *s*_*t*_, and the Critic network evaluates the value of this action; (3) Execution of feedback parameters: the feedback parameter λ_*t*_ is applied to the experts identified for adjustment by the recognition rule, generating personalized adjustment suggestions; (4) Expert opinion update: experts make gradual opinion adjustments according to the feedback intensity, producing a new preference matrix; (5) Environmental state update: the system recalculates the group consensus level and generates the new state *s*_(*t*+1)_; (6) Reward calculation: the reward *R*_*t*_ = ω_*c*_·*R*_*c*_+ω_*e*_·*R*_*e*_−ω_*l*_·*R*_*l*_ is computed based on consensus improvement, preference preservation, and the number of discussion rounds; (7) Policy optimization: the Critic network is updated via TD error, and the Actor network is updated via policy gradient. This closed-loop mechanism realizes an adaptive feedback cycle of “state perception—policy learning—opinion adjustment—environment update.”

In practical case applications, it is only necessary to input the distance vector*s*_*t*_ of the large group in real time. Once training is completed, the policy network can generate adaptive feedback parameters δ_*t*_ in real time according to the group state, thereby improving the efficiency of consensus adjustment and achieving data-driven adaptive machine-assisted collaborative decision-making.

### 3.5 Determination of attribute weights and alternative selection

#### 3.5.1 Acquisition of attribute weights

In this paper, the “Octopus” web crawler software is employed to collect data related to the “Beijing-Tianjin-Hebei rainstorm” from Weibo during a specific time period. Python software is then utilized to preprocess the data, and the *TF*−*IDF* algorithm is applied to analyze the processed content to obtain keyword term frequencies and inverse document frequencies. Subsequently, the keywords are matched with the attributes to derive the corresponding attributes and their weights. As a method widely used to evaluate the importance of feature terms in documents, the *TF*−*IDF* algorithm is computed as follows:


$$\begin{array}{l} TF-IDF=tf_{i,j},idf_{i}=\frac{n_{i,j}}{\sum_{k}n_{k,j}}\cdot \log\frac{\left|D\right|}{1+|j:t_{i}\in d_{j}|} \end{array}$$ (29)

where *tf*_*i, j*_ denotes the frequency of keyword *t*_*i*_in document *d*_*j*_, and the inverse document frequency *idf*_*i*_ measures the generality of the keyword, i.e., if a term appears in numerous documents, it is considered relatively common and of low importance; otherwise, it is deemed highly important. Here, *n*_*i, j*_ and ∑_*k*_*n*_*k, j*_ denote the number of occurrences of keyword *t*_*i*_ in document *d*_*j*_ and the total number of keywords in document *d*_*j*_, respectively. |*D*| denotes the total number of documents, and |*j*:*t*_*i*_ ∈ *d*_*j*_| denotes the number of documents containing keyword *t*_*i*_.

Given the attribute set *c*_*j*_(*j* = 1, 2, …, *N*) and the keyword set *kc*_*i*_(*i* = 1, 2, …, *n*) under attribute *c*_*j*_, the attribute weights are obtained by computing the *TF*−*IDF* values of the keywords, as shown in the following formula:


$$\begin{array}{l} w\left(c_{j}\right)=\underset{kc_{i}\in c_{j}}{\sum }{\left(TF-IDF\right)}_{kc_{i}} \end{array}$$ (30)

where (*TF*−*IDF*)_*k*_*c*__*i*__ denotes the TF-IDF value of keyword *kc*_*i*_.

After normalization, the standardized decision attribute weights are obtained as follows:


$$\begin{array}{l} w_{j}=\frac{w\left(c_{j}\right)}{\sum_{j=1}^{N}w\left(c_{j}\right)} \end{array}$$ (31)

#### 3.5.2 Alternative selection

After the group reaches consensus, the group decision matrix *G* = {_*g*_*lj*_}*P*×*N*_ is obtained. Given the attribute weight vector *w* = {*w*_*j*_|*j* = 1, 2, …, *N*} with $\sum_{j=1}^{N}w_{j}=1$ the comprehensive score of each alternative is computed as follows:


$$\begin{array}{l} O_{l}=\overset{N}{\underset{j}{\sum }}w_{j}\cdot g_{lj} \end{array}$$ (32)

The comprehensive score of each alternative is calculated using Equation 32, and the alternative with the highest score is selected as the optimal alternative.

In summary, the framework of the human-machine collaborative large-group feedback mechanism and consensus method based on the reinforcement learning algorithm is illustrated in Figure 2. The specific steps are as follows:

**Figure 2:**
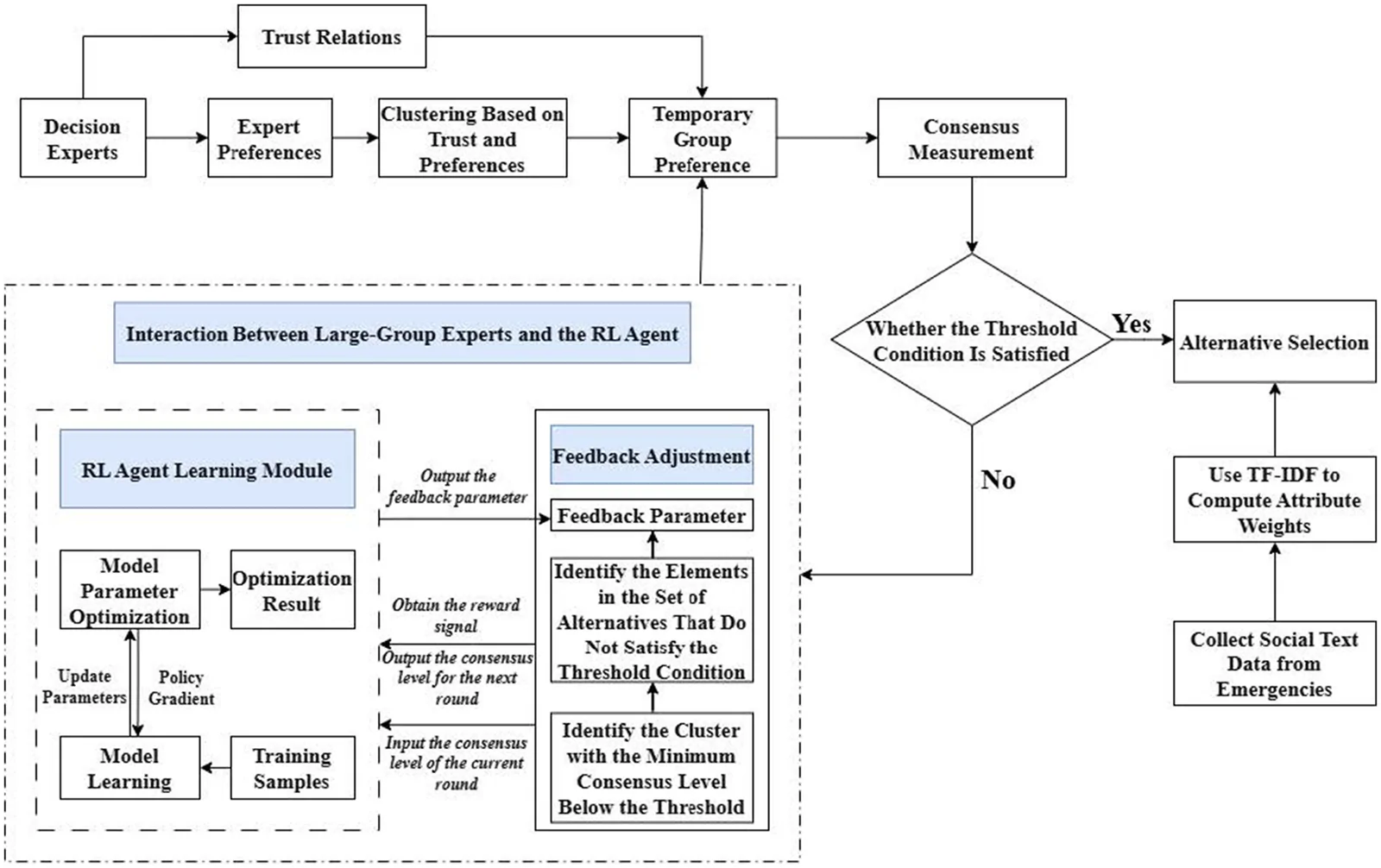
Framework of the adaptive feedback consensus method based on reinforcement learning (DDPG).

**Step 1: Acquisition of expert preferences and social relations**. The expert preferences in the form of hesitant fuzzy linguistic term sets are converted into numerical forms using Equations 1–5, and social network analysis is conducted based on the experts' social structure. Expert weights are then obtained using Equations 13, 14.

**Step 2: Large-group clustering**. The expert preferences and trust relations are integrated and clustered using the K-Means method to form*K*clusters. Subsequently, cluster weights are obtained using Equations 11, 12, and the temporary group preference is derived using Equations 15, 16.

**Step 3: Consensus measurement**. The initial consensus levels of each cluster and the group are calculated using Equations 17–21. To ensure both decision quality and timeliness, the group consensus threshold is set to γ = 0.9 (Tang and Liao, 2021). If the group consensus level does not reach the consensus threshold, the process proceeds to Step 4 for preference adjustment until the threshold is satisfied; otherwise, it proceeds to Step 5.

**Step 4: Feedback adjustment**. The expert preferences requiring adjustment are identified using Equations 25, 26. The feedback parameter is determined via the reinforcement learning algorithm, and the expert opinions are modified using Equation 26 until the group consensus threshold is met.

**Step 5: Alternative selection**. The final group preference matrix is obtained using Equation 16. Combined with public information, the attribute weights are derived, and the comprehensive score of each alternative is calculated using Equation 34. The alternative with the highest score is selected as the optimal alternative.

## 4 Case application

### 4.1 Case background

On July 29, 2023, Typhoon Doksuri moved northward carrying abundant moisture, triggering historically rare and extreme torrential rainfall in the Beijing-Tianjin-Hebei region. Heavy to extremely heavy rainfall occurred in most parts of Beijing, Tianjin, and central and southern Hebei, with some areas experiencing extraordinary downpours. Multiple departments and regions initiated emergency responses and carried out rescue operations. According to statistics, as of 24:00 on August 8, the disaster had caused 33 deaths and 18 missing persons in Beijing (Xinhua News Agency, 2023); across the entire Beijing-Tianjin-Hebei region, the typhoon claimed at least 21 lives, forced more than one million people to evacuate, and affected as many as 600,000 people [PNN (Public Television News Network), 2023].

In the aftermath of the disaster, the Central Committee of the Communist Party of China and the State Council attached great importance to the situation, emphasizing that the safety of people's lives and property must always be prioritized, and demanding the conscientious implementation of various flood prevention and disaster relief measures. The emergency management department promptly established an emergency decision-making rescue command center and convened a total of 20 experts from various fields, denoted as the expert set Ω = {*e*_*i*_|*i* = 1, 2, …, 20}. Based on the attribute set *C* = {*c*_1_, *c*_2_, *c*_3_, *c*_4_}, these experts were tasked with making decisions on four emergency alternatives *X* = {*x*_1_, *x*_2_, *x*_3_, *x*_4_} that were quickly formulated according to the actual situation, with the consensus threshold set at γ = 0.9 (Tang and Liao, 2021). The attributes include personnel search and rescue (*c*_1_), resource allocation and resettlement (*c*_2_), public opinion management (*c*_3_), and timeliness (*c*_4_). The four alternatives are as follows:

Alternative *x*_1_: Grasp the real-time situation, conduct headcounts as needed, check for injured or missing persons and verify their identities, and ensure that medical teams promptly treat critically and urgently injured individuals. Establish material distribution points and allocate daily necessities reasonably. For the core disaster-affected areas, organize water rescue (specialized rescue teams) to actively rescue people in distress and search for missing persons, and promptly release information on the disaster situation.

Alternative *x*_2_: Actively rescue surrounding trapped personnel, organize the safe transfer of people in hazardous areas, ascertain the number and identities of missing persons, and communicate with their families in a timely manner. Conduct public opinion guidance across various social media platforms, hold press conferences to alleviate public panic, and provide comfort to evacuees to ease their distress. Coordinate and organize all parties to reasonably allocate materials.

Alternative *x*_3_: Promptly assess the damage to transportation, communications, power supply, water supply, and medical facilities. The transportation department must repair damaged roads without delay to ensure that rescue teams and equipment can reach the disaster sites safely. Restore traffic safety as soon as possible to ensure the smooth progress of relief efforts. Dispatch various rescue teams to the affected areas to expand the scope of search and rescue operations. The logistics support department must ensure that relief supplies are delivered to stranded individuals in a timely manner, and hold regular press conferences to provide reassurance to all stakeholders.

Alternative *x*_4_: Organize water rescue (specialized rescue teams) to actively rescue people stranded in water, on rooftops, and in other dangerous environments. Dispatch engineering teams to carefully inspect dams, roads, bridges, and other structures for potential collapse risks. Identify and eliminate hazardous operations, and effectively manage secondary disasters such as hazardous chemical leaks. Allocate donated supplies and government-allocated materials in a reasonable manner, and promptly release updates on search and rescue progress and the disaster situation.

### 4.2 Decision-making process

According to the above decision-making procedure, the specific steps are as follows:

To validate the effectiveness of the proposed method, this paper constructs experimental data by simulating expert decision-making scenarios, and sets the evaluation attributes and alternatives with reference to actual emergency decision-making processes.

**Step 1:** Twenty experts express their preferences on four alternatives with respect to four attributes using multi-granularity hesitant fuzzy linguistic terms, and the evaluation matrices are obtained as shown in Appendix A. The linguistic term sets and their corresponding semantics adopted by the experts are presented in Table 1.

**Table 1:** Linguistic term sets and their corresponding semantics.

Granularity	Linguistic term set	Corresponding semantics
3	$S_{3}=\left\{S_{0}^{3},S_{1}^{3},S_{2}^{3}\right\}$	{Poor, Fair, Good}
5	$S_{5}=\left\{S_{0}^{5},S_{1}^{5},S_{2}^{5},S_{3}^{5},S_{4}^{5}\right\}$	{VeryPoor, Poor, Fair, Good, VeryGood}
7	$S_{7}=\left\{\begin{array}{c} s_{0}^{7},s_{1}^{7},s_{2}^{7},s_{3}^{7},s_{4}^{7},s_{5}^{7},s_{6}^{7} \end{array}\right\}$	$\left\{\begin{array}{c} \text{Very Poor},\text{Poor},\text{Relatively}\\ \text{Poor},\text{Fair},\\ \text{Relatively}\text{Good},\text{Good},\\ \text{Very}\text{Good} \end{array}\right\}$

Subsequently, the social trust relations among the experts are collected. The preference matrix of expert *e*_1_ is presented as follows:


$$\begin{array}{l} H^{1}=\left\{\begin{array}{cccc} \left\{s_{2}^{3}\right\} & \left\{s_{0}^{3},s_{1}^{3}\right\} & \left\{s_{1}^{3},s_{2}^{3}\right\} & \left\{s_{0}^{3}\right\}\\ \left\{s_{2}^{7},s_{3}^{7}\right\} & \left\{s_{5}^{7},s_{6}^{7}\right\} & \left\{s_{2}^{7},s_{3}^{7},s_{4}^{7}\right\} & \left\{s_{4}^{7},s_{5}^{7},s_{6}^{7}\right\}\\ \left\{s_{0}^{5},s_{1}^{5}\right\} & \left\{s_{2}^{5},s_{3}^{5},s_{4}^{5}\right\} & \left\{s_{3}^{5},s_{4}^{5}\right\} & \left\{s_{4}^{5}\right\}\\ \left\{s_{3}^{7}\right\} & \left\{s_{4}^{7},s_{5}^{7},s_{6}^{7}\right\} & \left\{s_{4}^{7}\right\} & \left\{s_{1}^{7},s_{2}^{7}\right\} \end{array}\right\} \end{array}$$ (33)

The score function values of the preference matrix are computed using Equations 1–4 and subsequently normalized to obtain the numerical preference evaluation values, as presented in Equation 34:


$$\begin{array}{l} V^{1}=\left[\begin{array}{cccc} 1 & 0.0469 & 0.4219 & 0\\ 0.3724 & 0.8307 & 0.4080 & 0.6959\\ 0.0820 & 0.5306 & 0.738 & 1\\ 0.5 & 0.5519 & 0.6667 & 0.2196 \end{array}\right] \end{array}$$ (34)

Additionally, the social network relations among the experts are obtained. The trust relation network is presented in Figure 3.

**Figure 3:**
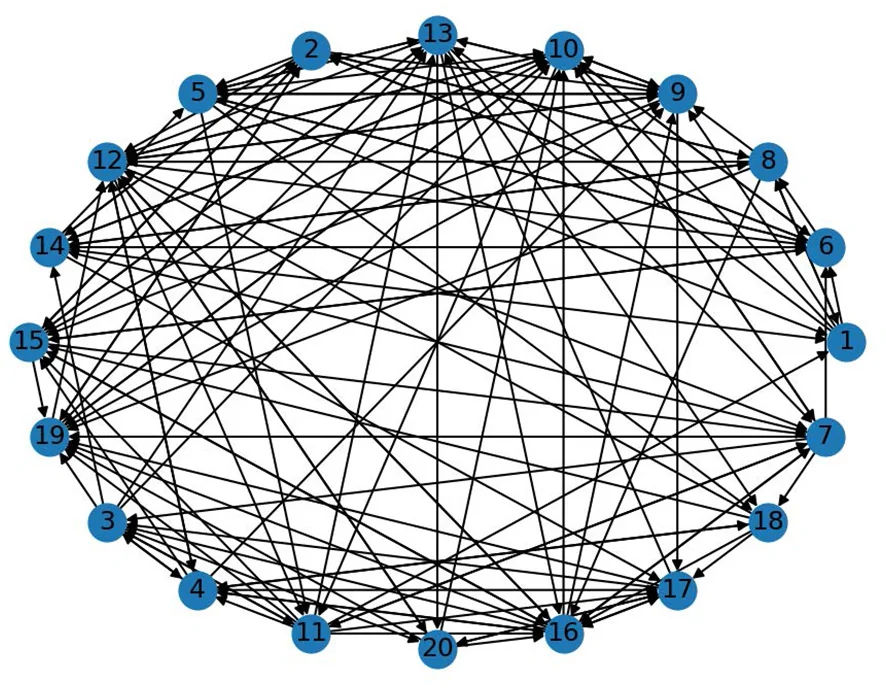
Social network relations among experts.

By employing Equations 6, 7, 13, 14, the expert weights are obtained, as presented in Table 2.

**Table 2:** Expert weight information.

Expert	*IND*_*i*_	*OUT*_*i*_	*TD*_*i*_	*W*_*i*_	Expert	*IND*_*i*_	*OUT*_*i*_	*TD*_*h*_	*W*_*i*_
*e*_1_	0.0074	0.0178	0.0526	0.019	e_11_	0.057	0.0326	0.4053	0.083
*e*_2_	0.0156	0.037	0.1105	0.04	e_12_	0.0378	0.0444	0.2684	0.071
*e*_3_	0.0141	0.0363	0.1	0.037	e_13_	0.0215	0.023	0.1526	0.038
*e*_4_	0.0133	0.0252	0.0947	0.03	e_14_	0.0356	0.0378	0.2526	0.064
*e*_5_	0.017	0.0244	0.1211	0.034	e_15_	0.0333	0.0415	0.2368	0.064
*e*_6_	0.0244	0.0252	0.1737	0.042	e_16_	0.0252	0.017	0.1789	0.037
*e*_7_	0.0304	0.0356	0.2158	0.056	e_17_	0.0156	0.0163	0.1105	0.027
*e*_8_	0.0163	0.0267	0.1158	0.034	e_18_	0.0593	0.0274	0.4211	0.08
*e*_9_	0.0578	0.0319	0.4105	0.083	e_19_	0.0141	0.0222	0.1	0.029
*e*_10_	0.0689	0.0274	0.4895	0.091	e_20_	0.057	0.0326	0.4053	0.083

**Step 2:** Based on the expert preference matrices and trust relations, the opinion similarity between experts is calculated using Equations 9 and 10. Subsequently, by further incorporating the trust relations among experts, the K-Means clustering method is applied. The clustering results are presented in Table 3.

**Table 3:** Clustering results.

Cluster(*C*^*k*^)	Size	Experts included	Weight
*C* ^1^	5	*e*_1_, *e*_2_, *e*_5_, *e*_8_, *e*_14_	0.1827
*C* ^2^	3	*e*_6_, *e*_10_, *e*_19_	0.2021
*C* ^3^	7	*e*_3_, *e*_4_, *e*_11_, *e*_16_, *e*_17_, *e*_18_, *e*_20_	0.3090
*C* ^4^	3	*e*_7_, *e*_13_, *e*_15_	0.1699
*C* ^5^	2	*e*_9_, *e*_12_	0.1363

By applying Equations 15 and 16, the temporary group preference is obtained, as presented in Equation 35:


$$\begin{array}{l} G=\left[\begin{array}{cccc} 0.2615 & 0.3156 & 0.4826 & 0.6203\\ 0.3327 & 0.4081 & 0.4973 & 0.6078\\ 0.4283 & 0.4125 & 0.4924 & 0.5829\\ 0.3646 & 0.5364 & 0.7008 & 0.6185 \end{array}\right] \end{array}$$ (35)

**Step 3:** Based on Equations 17–21, the initial consensus levels of each cluster and the group are calculated, as presented in Table 4.

**Table 4:** Initial consensus levels.

*CLC* ^1^	*CLC* ^2^	*CLC* ^3^	*CLC* ^4^	*CLC* ^5^	*GCL*
0.8489	0.8487	0.8795	0.8566	0.8511	0.8599

Since the group consensus level *GCL* = 0.8599 < γ = 0.9 is lower than the consensus threshold γ = 0.9, the group consensus level does not meet the predefined threshold, the consensus reaching process (CRP) is required to be performed.

**Step 4:** By applying Equations 25, 26 and the proposed consensus method, the feedback adjustment and consensus reaching process are conducted. After 6 iterations, the consensus threshold is satisfied. The feedback parameter results are presented in Table 5, the consensus results are presented in Table 6, and the group preference matrix is presented in Equation 36.


$$\begin{array}{l} G=\left\{\begin{array}{cccc} 0.2843 & 0.3362 & 0.4568 & 0.6301\\ 0.3211 & 0.4162 & 0.5055 & 0.5975\\ 0.4123 & 0.4170 & 0.5016 & 0.5622\\ 0.3751 & 0.5364 & 0.7155 & 0.651 \end{array}\right\} \end{array}$$ (36)

**Table 5:** Feedback parameter results.

Round (*t*)	1	2	3	4	5	6
Feedback parameter (δ_*t*_)	0.2195	0.2595	0.3685	0.3733	0.4878	0.5946

**Table 6:** Group consensus level iteration results.

Round (*t*)	1	2	3	4	5	6
GCL	0.8659	0.8720	0.8783	0.8851	0.8951	0.9043

**Step 5:** More than 50,000 posts related to the “Beijing-Tianjin-Hebei rainstorm” are collected from Weibo using the “Octopus” web crawler software. After data preprocessing, the attribute weights are obtained via the *TF*−*IDF* algorithm, as presented in Table 7.

**Table 7:** Attribute weights derived from Weibo data via the TF-IDF algorithm.

Attribute	Keywords	Weight
Personnel search and rescue	PLA, armed police, search and rescue, fire brigade, rescue, medical treatment, …	0.3051
Resource allocation and resettlement	Supplies, resettlement work, settlement, relief supplies, distribution, …	0.2119
Timeliness	Emergency, urgent, fastest, rapid, wartime, rescue, response, …	0.2288
Public opinion management	Information, early warning, release, latest updates, official, CCTV, rumor, …	0.2542

By applying Equation 34, the comprehensive scores of the four alternatives are calculated and ranked as *O*_4_>*O*_3_>*O*_2_>*O*_1_, which indicates that *x*_4_ is the optimal alternative.

### 4.3 Comparative analysis

To verify the feasibility and superiority of the refined score function, a comparison is conducted between the refined score function (which incorporates the hesitation degree) and the original score function. The comparative results are presented in Table 8.

**Table 8:** Comparison of the refined score function and the original score function.

Method / Score function	*O*_1_	*O*_2_	*O*_3_	*O*_4_	Ranking result
Refined score function	0.4227	0.4512	0.4718	0.5573	*x*_4_>*x*_3_>*x*_2_>*x*_1_
Original score function	0.4476	0.4737	0.5257	0.5713	*x*_4_>*x*_3_>*x*_1_>*x*_2_

From the ranking results, it can be observed that although the top two alternatives remain in the same order, the ranking order of the third and fourth alternatives differs. This indicates that the hesitation degree information embedded in the original decision matrix exerts an influence on the final ranking of alternatives. Moreover, under the refined score function incorporating the hesitation degree, the final scores of all alternatives are lower than those obtained by the original method. This is because, when converting expert preferences into numerical forms with the hesitation degree taken into account, each element in the expert decision matrix is multiplied by a real number not greater than 1, which consequently reduces the resulting values and thus lowers the final scores of the alternatives. This further demonstrates that the hesitation degree information affects the final ranking of alternatives. Therefore, it is necessary to incorporate the hesitation degree information, which substantiates the effectiveness and superiority of the proposed method.

To further evaluate the performance of the proposed feedback parameter adjustment method, it is compared with the methods in Xu and Xiao (2023) and Xu et al. (2015). The comparison results are presented in Figure 4.

**Figure 4:**
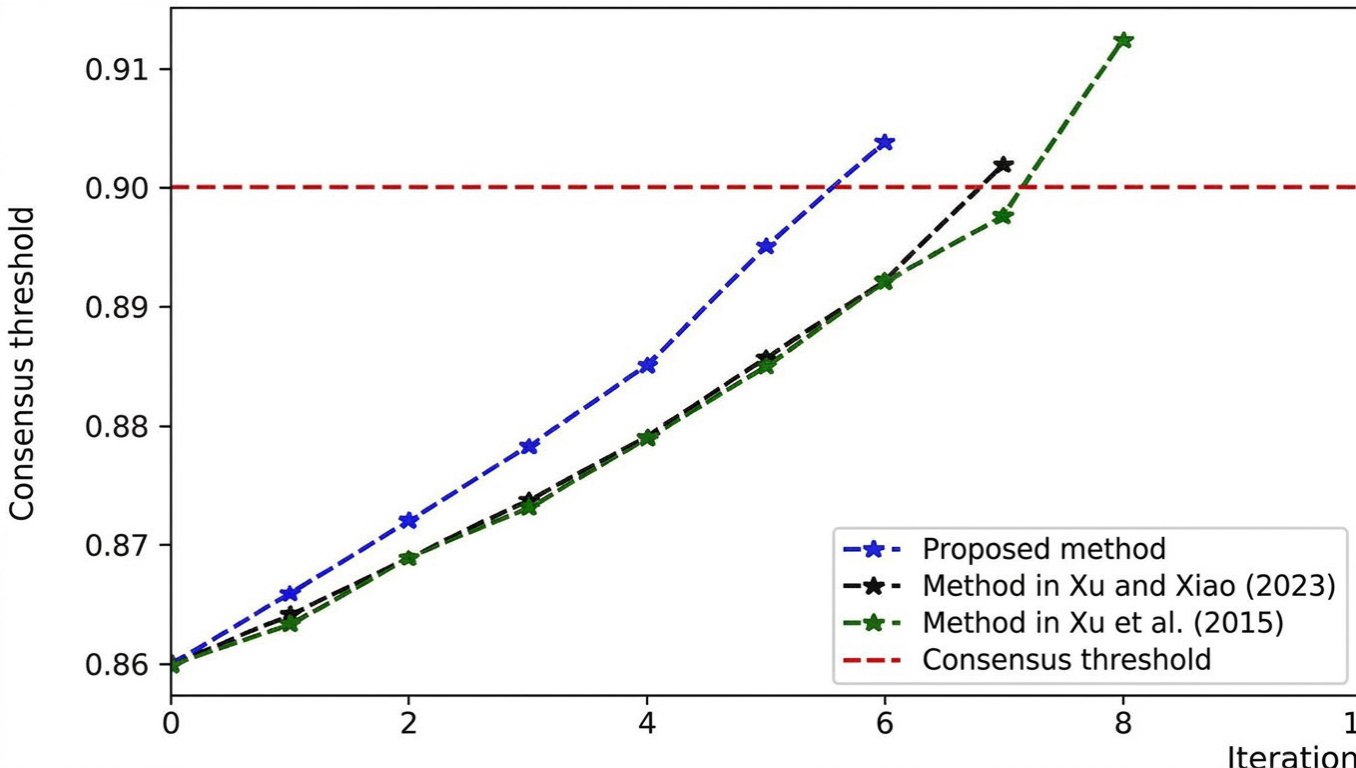
Comparison results of different methods.

In addition, the comparison results of different methods in terms of discussion rounds and alternative ranking are presented in Table 9.

**Table 9:** Comparison results of different methods.

Method	Discussion rounds	Adjustment parameter determination	Ranking result
Method in Xu and Xiao (2023)	7	Determined objectively	*x*_4_>*x*_3_>*x*_2_>*x*_1_
Method in Xu et al. (2015)	8	Combined subjective and objective determination	*x*_4_>*x*_2_>*x*_3_>*x*_1_
Proposed method	6	Determined objectively	*x*_4_>*x*_3_>*x*_2_>*x*_1_

From the final results, it can be observed that both the proposed method and the compared methods identify *x*_4_ and *x*_1_ as the optimal and worst alternatives, respectively, which demonstrates the rationality of the proposed method. In terms of the determination of adjustment parameters, the method in Xu et al. (2015) obtains a comprehensive adjustment parameter by combining subjective and objective determinations. In contrast, both the method in Xu and Xiao (2023) and the proposed method determine the adjustment parameter objectively. Notably, the subjective adjustment parameter in Xu et al. (2015) requires expert discussion, which may lead to unreliable decision outcomes, and the number of discussion rounds is significantly influenced by the subjective parameter. Objectively determined adjustment parameters, on the other hand, are typically based on stable data and standards, which can reduce the impact of subjective bias on decision results.

Comparing the method in Xu and Xiao (2023) with the proposed method, the ranking results of all alternatives are identical, but the method in Xu and Xiao (2023) requires more discussion rounds than the proposed method. Specifically, the method in Xu and Xiao (2023) determines the feedback parameter based on trust relations and similarity relations, whereas the proposed method employs a reinforcement learning algorithm. By effectively learning from historical experience and continuously improving through interaction with the decision-making environment, the reinforcement learning algorithm ensures the efficiency and correctness of the feedback adjustment process.

Comparing the method in Xu et al. (2015) with the proposed method, although the optimal and worst alternatives are ranked identically, the overall rankings of the alternatives differ. This is because the parameter determination in Xu et al. (2015) involves subjectivity, and the resulting discussion rounds are also greater than those of the proposed method. In summary, the proposed method exhibits strong superiority.

## 5 Extended experiments and analysis

To further validate the effectiveness and robustness of the proposed method, extended experiments are carried out in this section on the basis of the original case analysis, encompassing consensus experiments with varying numbers of experts, parameter sensitivity analysis, and comparative experiments with other methods.

### 5.1 Description of algorithm parameter settings

To ensure the reproducibility of the experimental results, this section provides a detailed description of the key algorithm parameters of the proposed method and the rationale behind their configuration. All parameters are determined via preliminary experiments, and their settings are validated to ensure algorithm convergence and stability.

(1) **DDPG algorithm parameter settings**: The Actor network learning rate is set to *lr*_*c*_ = 0.001, following common practice in reinforcement learning to ensure stable policy gradient updates. The Critic network learning rate is set to *lr*_*c*_ = 0.002, slightly higher than the Actor's learning rate to accelerate the convergence of the value function. The discount factor is γ = 0.95, balancing immediate rewards and long-term returns. The replay buffer size is *N* = 1, 000, ensuring diversity of training samples. The mini-batch size is *batch*_*size* = 64, improving training efficiency. The exploration noise standard deviation is σ = 0.05, initially set larger to encourage exploration and gradually decayed to promote exploitation. The soft update coefficient is τ = 0.001, enabling smooth tracking of the target network parameters.

The above parameter settings refer to the recommended configurations in Hassani et al. (2023) and Lillicrap et al. (2016), and are further fine-tuned via grid search over the range [0.0005,0.005] × [0.001,0.01]. The configuration that yields the fastest training convergence is finally selected.

(2) **Model parameter settings**: The consensus threshold is set to δ = 0.9, as recommended by Xu et al. (2015) for emergency decision-making scenarios. The fusion coefficient is set to θ = 0.5, determined via sensitivity analysis (see Section 5.3) that equal weighting of preference similarity and trust relationship yields the optimal performance. The number of K-Means clusters is determined by searching over the range [2,8] using the silhouette coefficient. The reward function weights are set as ω_*c*_ = 0.5,ω_*e*_ = 0.3, and ω_*l*_ = 0.2, corresponding to the consensus improvement reward, preference preservation reward, and round penalty, respectively. Through sensitivity analysis (see Section 5.3), the default configuration is determined to achieve the best balance between consensus efficiency and preference preservation.

### 5.2 Experiments with expert groups of different sizes

To verify the applicability of the proposed method in large-scale group decision-making scenarios with different group sizes, simulated decision scenarios involving 20, 50, 100, and 200 experts are, respectively, established, with the consensus threshold set to 0.9. Each experiment is repeated 10 times and the results are averaged. The experimental results are shown in Table 10.

**Table 10:** Experimental results of expert groups with different sizes.

Number of experts	Initial consensus	Rounds to consensus	Final consensus	Preference preservation rate	Number of clusters
20	0.8567	5.6	0.9076	0.7173	3
50	0.8506	5.9	0.9081	0.7003	4
100	0.8405	6.4	0.9091	0.6924	5
200	0.8322	7.5	0.9102	0.6802	6

As shown in Table 10, as the number of experts increases, the initial consensus level decreases slightly (from 0.8567 to 0.8322), and the number of rounds required to reach consensus increases accordingly (from 5.6 to 7.5 rounds). However, the consensus threshold of 0.9 can be achieved within 8 rounds across all group sizes. The preference preservation rate remains between 0.68 and 0.72, indicating that the DDPG agent can effectively balance consensus efficiency and expert opinion retention under different group sizes. This verifies the applicability and stability of the proposed method in large-scale group decision-making scenarios with varying group sizes.

To further validate the generalization ability of the proposed method, additional experiments are designed using different types of simulated datasets. Specifically, three types of preference distributions are considered: (1) Uniform distribution dataset: expert preferences are randomly generated from a uniform distribution *U*(0,1), simulating scenarios without obvious preference clustering; (2) Normal distribution dataset: expert preferences are randomly generated from a normal distribution *N*(0.5,0.15), simulating scenarios with a mainstream opinion and moderate divergence; (3) Multimodal distribution dataset: expert preferences are randomly generated from a Gaussian mixture model 0.4*N*(0.3,0.08) + 0.6*N*(0.7,0.08), simulating scenarios with clear opinion polarization. The three datasets are tested with expert group sizes of 50, 100, and 200, respectively. Experimental results show that the proposed method can reach the consensus threshold of 0.9 within 8 rounds under all three distribution types, with the preference preservation rate ranging from 0.65 to 0.73. Among them, the multimodal distribution scenario requires slightly more rounds to reach consensus (approximately 0.5–1.0 rounds more on average), but still within an acceptable range, which confirms the adaptability and generalization ability of the proposed method to different data distributions.

### 5.3 Parameter sensitivity analysis

(1) **Sensitivity analysis of the fusion coefficient** **θ**. Taking 50 experts as an example, the clustering and consensus performance are tested for θ values of 0.0, 0.3, 0.5, 0.7, and 1.0. The results are shown in Table 11.

**Table 11:** Sensitivity analysis results of the fusion coefficient θ.

θ value	Rounds to consensus	Final consensus	Preference preservation rate	Silhouette coefficient
0.0	8.0	0.8881	0.6694	0.1228
0.3	7.2	0.8962	0.7000	0.2812
0.5	6.2	0.9091	0.7056	0.4308
0.7	7.0	0.9040	0.7161	0.3497
1.0	9.0	0.8910	0.7214	0.2348

As shown in Table 11, when θ = 0.5, the silhouette coefficient is the highest (0.4308), the number of rounds required to reach consensus is the smallest (6.2 rounds), and the final consensus level is the highest (0.9091), indicating that equal weighting of preference similarity and trust relationships yields the best clustering performance. When θ = 0.0 (trust relationships only) or θ = 1.0 (preference similarity only), both clustering effectiveness and consensus efficiency decrease significantly, confirming the necessity of integrating the two types of information.

(2) **Sensitivity analysis of DDPG training parameters**. The effects of different Actor/Critic learning rates and exploration noise on consensus performance are tested, and the results are shown in Table 12.

**Table 12:** Sensitivity analysis results of DDPG training parameters.

Parameter configuration	Rounds to consensus	Final consensus	Preference preservation rate
*lr*_*a*_ = 0.0005, *lr*_*c*_ = 0.001, σ = 0.03	6.0	0.9069	0.7057
*lr*_*a*_ = 0.001, *lr*_*c*_ = 0.002, σ = 0.05	6.4	0.9068	0.7068
*lr*_*a*_ = 0.002, *lr*_*c*_ = 0.004, σ = 0.10	7.4	0.9060	0.6793
*lr*_*a*_ = 0.005, *lr*_*c*_ = 0.01, σ = 0.15	11.8	0.9082	0.6845

As shown in Table 12, when the learning rate is relatively small (*lr*_*a*_ = 0.0005), the training process is stable, the number of rounds required to reach consensus is the smallest (6.0 rounds), and the preference preservation rate is relatively high (0.7057). However, an excessively small learning rate may lead to insufficient parameter updates, resulting in a slight increase in the number of rounds needed to reach consensus under the multimodal distribution experiment (approximately 0.5–1.0 additional rounds on average), and the adaptability to extreme data distributions is slightly weaker. When the learning rate is moderate (*lr*_*a*_ = 0.001), the number of rounds increases slightly (6.4 rounds), but the preference preservation rate is the highest (0.7068), and the training process is more robust with lower sensitivity to the initial random seed. When the learning rate is too large (*lr*_*a*_ = 0.005), the training exhibits noticeable fluctuations, with the number of rounds significantly increasing (11.8 rounds) and the preference preservation rate dropping to 0.6845, indicating that an excessive learning rate leads to policy oscillation. Considering convergence speed, stability, and generalization ability comprehensively, this paper selects *lr*_*a*_ = 0.001, *lr*_*c*_ = 0.002, σ = 0.05 as the default configuration, which ensures training stability while maintaining good adaptability across scenarios.

(3) **Sensitivity analysis of reward weights**. The effects of different reward weight configurations on consensus performance are tested, and the results are shown in Table 13.

**Table 13:** Sensitivity analysis results of reward weights.

Configuration	ω_*c*_, ω_*e*_, ω_*l*_	Rounds to consensus	Preference preservation rate
Default	0.5, 0.3, 0.2	6.0	0.6975
Consensus-oriented	0.6, 0.2, 0.2	5.8	0.6824
Preference-oriented	0.4, 0.4, 0.2	7.0	0.7235
Preference-first	0.5, 0.2, 0.3	5.7	0.6885
Strong consensus-driven	0.7, 0.2, 0.1	5.2	0.6498

As shown in Table 13, different reward weight configurations have significant impacts on both consensus efficiency and preference preservation rate. When ω_*c*_ increases (consensus-oriented), the number of rounds decreases but the preference preservation rate declines. When ω_*e*_ increases (preference-oriented), the preference preservation rate improves but the number of rounds increases. The default configuration (ω_*c*_ = 0.5, ω_*e*_ = 0.3, ω_*l*_ = 0.2) achieves a good balance between the two objectives.

### 5.4 Method comparison experiments

To verify the superiority of the proposed DDPG-based adaptive feedback method, it is compared with a fixed-parameter feedback method (λ = 0.3), a non-DDPG feedback method (random small-step adjustment), and a traditional CRP method (linear increasing parameters). Experiments are conducted with group sizes of 50, 100, and 200 experts, each repeated 10 times. The evaluation metrics include rounds to consensus, maximum consensus level, expert preference preservation rate, and time complexity. The comparison results are presented in Table 14.

**Table 14:** Comparative experimental results of different methods.

Number of Experts	Method	Rounds to consensus	Final consensus	Preference preservation rate	Time (s)
50	Proposed DDPG method	6.2	0.9090	0.6849	0.372
50	Fixed parameter (λ = 0.3)	8.4	0.9056	0.6306	0.504
50	Non-DDPG feedback	12.4	0.9045	0.7632	0.744
50	Traditional CRP method	10.5	0.9055	0.6075	0.630
100	Proposed DDPG method	6.5	0.9084	0.6990	0.715
100	Fixed parameter (λ = 0.3)	8.6	0.9046	0.6135	0.946
100	Non-DDPG feedback	13.3	0.9044	0.7592	1.463
100	Traditional CRP method	11.2	0.9048	0.5915	1.232
200	Proposed DDPG method	6.9	0.9092	0.6920	1.449
200	Fixed parameter (λ = 0.3)	9.5	0.9059	0.5948	1.995
200	Non-DDPG feedback	14.4	0.9041	0.7570	3.024
200	Traditional CRP method	12.1	0.9053	0.5821	2.541

As shown in Table 14, under all group sizes, the proposed DDPG method achieves the fewest rounds to consensus (6.2 rounds for 50 experts vs. 8.4 for fixed parameter, 12.4 for non-DDPG feedback, and 10.5 for traditional CRP), and its preference preservation rate outperforms both the fixed-parameter and traditional CRP methods. Although the non-DDPG feedback method yields a slightly higher preference preservation rate, it requires significantly more rounds to reach consensus (approximately twice as many as the proposed method) and incurs higher time complexity. This indicates that the DDPG agent can dynamically adjust feedback parameters according to the group state, effectively maintaining expert preferences while ensuring consensus efficiency. Moreover, as the expert group size increases, the advantages of the proposed method become more pronounced, further validating its applicability in large-scale group decision-making scenarios.

It should be further noted that there exists an inherent trade-off between consensus efficiency and expert preference preservation in the proposed method. As shown in Table 14, the non-DDPG feedback method achieves a slightly higher preference preservation rate than the proposed method (e.g., 0.7632 vs. 0.6849 for 50 experts). This is because the non-DDPG method adopts a conservative small-step random adjustment strategy, which results in smaller modifications to the original expert preferences. However, the number of rounds required to reach consensus is approximately twice that of the proposed method (12.4 rounds vs. 6.2 rounds). In emergency decision-making scenarios, where disaster conditions change rapidly and time is critical, an excessively long consensus process may lead to missing the optimal decision window. Therefore, the proposed method achieves a significant improvement in efficiency at the cost of moderate preference adjustments, demonstrating stronger practical value in emergency contexts. For decision tasks in non-urgent scenarios that require higher preference preservation, a more conservative feedback strategy can be realized by lowering the consensus improvement weight (ω_*c*_) or increasing the preference preservation weight (ω_*e*_) in the reward function. This flexibility is also a key advantage of the DDPG-based adaptive mechanism.

It should be further clarified that this paper compares the proposed method with four benchmark approaches: (1) Traditional CRP method (linearly increasing parameters): adopting the trust-based feedback mechanism from Xu and Xiao (2023) with parameters increasing linearly at a fixed rate; (2) Fixed-parameter feedback method (λ = 0.3): using the subjective-objective parameter determination approach from Xu et al. (2015) with a constant value of 0.3; (3) Non-DDPG feedback method (random small-step adjustment): without reinforcement learning, performing opinion adjustment with random small step sizes (λ ε [0.1, 0.3]); and (4) The proposed DDPG-based adaptive method. The addition of the non-DDPG feedback method and the traditional CRP method as baselines covers four distinct paradigms—rule-driven, fixed-parameter, random-adjustment, and reinforcement-learning-based—thereby enabling a more comprehensive validation of the proposed method's effectiveness. Furthermore, this paper evaluates all methods from four dimensions: rounds to consensus, final consensus level, preference preservation rate, and time complexity, ensuring the comprehensiveness and objectivity of the comparative results.

## 6 Conclusion

With the increasing demand for digital agile emergency response and the growing emphasis on collaborative intelligence synergy, the deep integration of machine intelligence's dynamic computational advantages with the structured experience of human experts has become a fundamental trend in the evolution of Large-Scale Group Decision-Making (LSGDM). To address the limitations of traditional consensus-reaching mechanisms—namely, information loss during preference transformation and the rigidity of discrete feedback parameters—this paper proposes a machine-assisted adaptive feedback consensus model based on deep reinforcement learning. The methodological innovations of this study are realized in the following three dimensions:

By incorporating the hesitation degree to improve the classical score function, the proposed approach compensates for the shortcoming of traditional Hesitant Fuzzy Linguistic Term Sets (HFLTS) which are prone to misjudgment due to equivalent values during uncertain preference conversion, thereby preserving the decision-making heterogeneity of experts to the greatest extent, and integrating with trust networks to achieve more robust complex social network clustering.

To overcome the inflexibility of predefined parameters in conventional rule-driven models, this paper proposes an adaptive feedback mechanism based on deep reinforcement learning. This mechanism reformulates the consensus-reaching process as a Markov Decision Process (MDP), in which the agent dynamically outputs continuous and flexible feedback parameters through the dual-network interaction framework of the DDPG algorithm. Experimental results demonstrate that this regulatory approach can effectively reduce over-adjustment and improve the preservation of expert opinions.

A multi-objective reward function is designed to achieve Pareto optimization of the dual objectives—”consensus convergence timeliness” and “preservation of individual expert opinions.” Meanwhile, attribute weights are obtained through text mining of external online big data (TF-IDF), forming a machine-assisted architecture that integrates “individual linguistic cognition—small-group network topology—large-group adaptive evolution—public social preference mapping.”

The real-world case study of the “Beijing-Tianjin-Hebei rainstorm” emergency decision-making, along with comparative experiments, demonstrates that the proposed model can drive high-dimensional heterogeneous experts to rapidly converge to high-quality consensus with fewer negotiation rounds.

It should be noted that the current model assumes that experts make gradual adjustments according to the feedback mechanism, with AI assisting in generating feedback strategies and experts adjusting their opinions based on the feedback intensity. Future research will further consider experts' active acceptance, rejection, partial adoption, and negotiation behaviors to construct a more realistic human-machine interactive decision model.

## Data Availability

The original contributions presented in the study are included in the article/supplementary material, further inquiries can be directed to the corresponding author.

## Appendix A

**Table A1:**

$H^{1}=\left[\begin{array}{cccc} \left\{s_{0}^{3},s_{1}^{3}\right\} & \left\{s_{0}^{3},s_{1}^{3}\right\} & \left\{s_{1}^{3},s_{2}^{3}\right\} & \left\{s_{2}^{3}\right\}\\ \left\{s_{1}^{5}\right\} & \left\{s_{1}^{5},s_{2}^{5},s_{3}^{5}\right\} & \left\{s_{1}^{5},s_{2}^{5},s_{3}^{5}\right\} & \left\{s_{4}^{5}\right\}\\ \left\{s_{1}^{7},s_{2}^{7}\right\} & \left\{s_{2}^{7},s_{3}^{7}\right\} & \left\{s_{3}^{7},s_{4}^{7}\right\} & \left\{s_{6}^{7}\right\}\\ \left\{s_{2}^{7},s_{3}^{7}\right\} & \left\{s_{3}^{7},s_{4}^{7}\right\} & \left\{s_{5}^{7}\right\} & \left\{s_{6}^{7}\right\} \end{array}\right]$	$H^{2}=\left[\begin{array}{cccc} \left\{s_{0}^{3},s_{1}^{3}\right\} & \left\{s_{1}^{3},s_{2}^{3}\right\} & \left\{s_{1}^{3},s_{2}^{3}\right\} & \left\{s_{2}^{3}\right\}\\ \left\{s_{1}^{5},s_{2}^{5}\right\} & \left\{s_{2}^{5}\right\} & \left\{s_{1}^{5},s_{2}^{5},s_{3}^{5}\right\} & \left\{s_{4}^{5}\right\}\\ \left\{s_{1}^{7},s_{2}^{7},s_{3}^{7}\right\} & \left\{s_{2}^{7},s_{3}^{7},s_{4}^{7}\right\} & \left\{s_{3}^{7},s_{4}^{7}\right\} & \left\{s_{6}^{7}\right\}\\ \left\{s_{2}^{7},s_{3}^{7}\right\} & \left\{s_{3}^{7},s_{4}^{7}\right\} & \left\{s_{5}^{7},s_{6}^{7}\right\} & \left\{s_{5}^{7},s_{6}^{7}\right\} \end{array}\right]$
$H^{3}=\left[\begin{array}{cccc} \left\{s_{1}^{3}\right\} & \left\{s_{1}^{3}\right\} & \left\{s_{1}^{3},s_{2}^{3}\right\} & \left\{s_{1}^{3},s_{2}^{3}\right\}\\ \left\{s_{0}^{5},s_{1}^{5},s_{2}^{5}\right\} & \left\{s_{1}^{5},s_{2}^{5},s_{3}^{5},s_{4}^{5}\right\} & \left\{s_{1}^{5},s_{2}^{5}\right\} & \left\{s_{3}^{5},s_{4}^{5}\right\}\\ \left\{s_{1}^{7},s_{2}^{7},s_{3}^{7}\right\} & \left\{s_{2}^{7},s_{3}^{7}\right\} & \left\{s_{2}^{7},s_{3}^{7},s_{4}^{7}\right\} & \left\{s_{4}^{7},s_{5}^{7},s_{6}^{7}\right\}\\ \left\{s_{2}^{7}\right\} & \left\{s_{3}^{7},s_{4}^{7},s_{5}^{7}\right\} & \left\{s_{4}^{7},s_{5}^{7}\right\} & \left\{s_{6}^{7}\right\} \end{array}\right]$	$H^{4}=\left[\begin{array}{cccc} \left\{s_{1}^{3}\right\} & \left\{s_{1}^{3}\right\} & \left\{s_{1}^{3},s_{2}^{3}\right\} & \left\{s_{2}^{3}\right\}\\ \left\{s_{1}^{5},s_{2}^{5}\right\} & \left\{s_{2}^{5},s_{3}^{5}\right\} & \left\{s_{1}^{5},s_{2}^{5}\right\} & \left\{s_{1}^{5},s_{2}^{5},s_{3}^{5},s_{4}^{5}\right\}\\ \left\{s_{1}^{7},s_{2}^{7},s_{3}^{7}\right\} & \left\{s_{2}^{7},s_{3}^{7},s_{4}^{7}\right\} & \left\{s_{3}^{7},s_{4}^{7},s_{5}^{7}\right\} & \left\{s_{4}^{7},s_{5}^{7}\right\}\\ \left\{s_{2}^{7},s_{3}^{7}\right\} & \left\{s_{3}^{7},s_{4}^{7}\right\} & \left\{s_{4}^{7},s_{5}^{7},s_{6}^{7}\right\} & \left\{s_{5}^{7}\right\} \end{array}\right]$
$H^{5}=\left[\begin{array}{cccc} \left\{s_{0}^{3},s_{1}^{3}\right\} & \left\{s_{1}^{3}\right\} & \left\{s_{1}^{3},s_{2}^{3}\right\} & \left\{s_{1}^{3},s_{2}^{3}\right\}\\ \left\{s_{2}^{5},s_{3}^{5},s_{4}^{5}\right\} & \left\{s_{1}^{5},s_{2}^{5},s_{3}^{5}\right\} & \left\{s_{1}^{5},s_{2}^{5},s_{3}^{5}\right\} & \left\{s_{2}^{5},s_{3}^{5},s_{4}^{5}\right\}\\ \left\{s_{2}^{7},s_{3}^{7},s_{4}^{7}\right\} & \left\{s_{0}^{7},s_{1}^{7},s_{2}^{7}\right\} & \left\{s_{3}^{7},s_{4}^{7}\right\} & \left\{s_{4}^{7},s_{5}^{7}\right\}\\ \left\{s_{1}^{7},s_{2}^{7}\right\} & \left\{s_{3}^{7},s_{4}^{7},s_{5}^{7}\right\} & \left\{s_{4}^{7},s_{5}^{7},s_{6}^{7}\right\} & \left\{s_{3}^{7},s_{4}^{7}\right\} \end{array}\right]$	$H^{6}=\left[\begin{array}{cccc} \left\{s_{0}^{3},s_{1}^{3}\right\} & \left\{s_{0}^{3},s_{1}^{3}\right\} & \left\{s_{1}^{3},s_{2}^{3}\right\} & \left\{s_{2}^{3}\right\}\\ \left\{s_{3}^{5},s_{4}^{5}\right\} & \left\{s_{1}^{5},s_{2}^{5},s_{3}^{5},s_{4}^{5}\right\} & \left\{s_{2}^{5},s_{3}^{5}\right\} & \left\{s_{2}^{5},s_{3}^{5}\right\}\\ \left\{s_{5}^{7},s_{6}^{7}\right\} & \left\{s_{2}^{7},s_{3}^{7}\right\} & \left\{s_{3}^{7},s_{4}^{7}\right\} & \left\{s_{6}^{7}\right\}\\ \left\{s_{0}^{3},s_{1}^{3}\right\} & \left\{s_{1}^{3}\right\} & \left\{s_{1}^{3},s_{2}^{3}\right\} & \left\{s_{1}^{3},s_{2}^{3}\right\} \end{array}\right]$
$H^{7}=\left[\begin{array}{cccc} \left\{s_{0}^{3},s_{1}^{3}\right\} & \left\{s_{0}^{3},s_{1}^{3}\right\} & \left\{s_{1}^{3},s_{2}^{3}\right\} & \left\{s_{2}^{3}\right\}\\ \left\{s_{1}^{3},s_{2}^{3}\right\} & \left\{s_{1}^{3},s_{2}^{3}\right\} & \left\{s_{2}^{3}\right\} & \left\{s_{1}^{3},s_{2}^{3}\right\}\\ \left\{s_{1}^{7},s_{2}^{7},s_{3}^{7},s_{4}^{7}\right\} & \left\{s_{2}^{7},s_{3}^{7},s_{4}^{7}\right\} & \left\{s_{2}^{7},s_{3}^{7},s_{4}^{7}\right\} & \left\{s_{3}^{7},s_{4}^{7},s_{5}^{7},s_{6}^{7}\right\}\\ \left\{s_{2}^{7},s_{3}^{7},s_{4}^{7},s_{5}^{7}\right\} & \left\{s_{3}^{7},s_{4}^{7},s_{5}^{7}\right\} & \left\{s_{5}^{7}\right\} & \left\{s_{1}^{7},s_{2}^{7},s_{3}^{7}\right\} \end{array}\right]$	$H^{8}=\left[\begin{array}{cccc} \left\{s_{1}^{3}\right\} & \left\{s_{0}^{3},s_{1}^{3}\right\} & \left\{s_{1}^{3},s_{2}^{3}\right\} & \left\{s_{1}^{3}\right\}\\ \left\{s_{1}^{5}\right\} & \left\{s_{0}^{5},s_{1}^{5},s_{2}^{5}\right\} & \left\{s_{1}^{5},s_{2}^{5},s_{3}^{5}\right\} & \left\{s_{2}^{5},s_{3}^{5}\right\}\\ \left\{s_{3}^{5},s_{4}^{5}\right\} & \left\{s_{0}^{5},s_{1}^{5},s_{2}^{5}\right\} & \left\{s_{2}^{5},s_{3}^{5}\right\} & \left\{s_{1}^{5},s_{2}^{5},s_{3}^{5},s_{4}^{5}\right\}\\ \left\{s_{2}^{7},s_{3}^{7},s_{4}^{7},s_{5}^{7},s_{6}^{7}\right\} & \left\{s_{3}^{7},s_{4}^{7}\right\} & \left\{s_{5}^{7}\right\} & \left\{s_{6}^{7}\right\} \end{array}\right]$
$H^{9}=\left[\begin{array}{cccc} \left\{s_{1}^{3},s_{2}^{3}\right\} & \left\{s_{1}^{3}\right\} & \left\{s_{1}^{3},s_{2}^{3}\right\} & \left\{s_{0}^{3},s_{1}^{3}\right\}\\ \left\{s_{1}^{7},s_{2}^{7},s_{3}^{7}\right\} & \left\{s_{4}^{7},s_{5}^{7},s_{6}^{7}\right\} & \left\{s_{3}^{7},s_{4}^{7},s_{5}^{7}\right\} & \left\{s_{2}^{7},s_{3}^{7}\right\}\\ \left\{s_{3}^{5}\right\} & \left\{s_{2}^{5},s_{3}^{5},s_{4}^{5}\right\} & \left\{s_{1}^{5},s_{2}^{5}\right\} & \left\{s_{2}^{5},s_{3}^{5},s_{4}^{5}\right\}\\ \left\{s_{2}^{7},s_{3}^{7},s_{4}^{7},s_{5}^{7}\right\} & \left\{s_{3}^{7},s_{4}^{7},s_{5}^{7},s_{6}^{7}\right\} & \left\{s_{5}^{7}\right\} & \left\{s_{2}^{7},s_{3}^{7},s_{4}^{7},s_{5}^{7}\right\} \end{array}\right]$	$H^{10}=\left[\begin{array}{cccc} \left\{s_{0}^{3},s_{1}^{3}\right\} & \left\{s_{1}^{3}\right\} & \left\{s_{2}^{3}\right\} & \left\{s_{1}^{3},s_{2}^{3}\right\}\\ \left\{s_{1}^{3}\right\} & \left\{s_{1}^{3}\right\} & \left\{s_{1}^{3}\right\} & \left\{s_{1}^{3},s_{2}^{3}\right\}\\ \left\{s_{1}^{3}\right\} & \left\{s_{1}^{3},s_{2}^{3}\right\} & \left\{s_{1}^{3}\right\} & \left\{s_{1}^{3},s_{2}^{3}\right\}\\ \left\{s_{1}^{3}\right\} & \left\{s_{1}^{3}\right\} & \left\{s_{1}^{3},s_{2}^{3}\right\} & \left\{s_{1}^{3}\right\} \end{array}\right]$
$H^{11}=\left[\begin{array}{cccc} \left\{s_{0}^{3}\right\} & \left\{s_{0}^{3},s_{1}^{3}\right\} & \left\{s_{1}^{3},s_{2}^{3}\right\} & \left\{s_{2}^{3}\right\}\\ \left\{s_{1}^{5},s_{2}^{5},s_{3}^{5}\right\} & \left\{s_{2}^{5},s_{3}^{5},s_{4}^{5}\right\} & \left\{s_{2}^{5},s_{3}^{5}\right\} & \left\{s_{2}^{5},s_{3}^{5},s_{4}^{5}\right\}\\ \left\{s_{1}^{7},s_{2}^{7}\right\} & \left\{s_{4}^{7},s_{5}^{7},s_{6}^{7}\right\} & \left\{s_{3}^{7}\right\} & \left\{s_{2}^{7},s_{3}^{7},s_{4}^{7}\right\}\\ \left\{s_{2}^{7},s_{3}^{7}\right\} & \left\{s_{3}^{7},s_{4}^{7},s_{5}^{7},s_{6}^{7}\right\} & \left\{s_{5}^{7},s_{6}^{7}\right\} & \left\{s_{2}^{7},s_{3}^{7},s_{4}^{7},s_{5}^{7}\right\} \end{array}\right]$	$H^{12}=\left[\begin{array}{cccc} \left\{s_{1}^{5},s_{2}^{5},s_{3}^{5}\right\} & \left\{s_{1}^{5},s_{2}^{5},s_{3}^{5}\right\} & \left\{s_{1}^{5},s_{2}^{5},s_{3}^{5}\right\} & \left\{s_{2}^{5},s_{3}^{5}\right\}\\ \left\{s_{0}^{5},s_{1}^{5}\right\} & \left\{s_{1}^{5},s_{2}^{5},s_{3}^{5},s_{4}^{5}\right\} & \left\{s_{1}^{5},s_{2}^{5},s_{3}^{5}\right\} & \left\{s_{2}^{5},s_{3}^{5},s_{4}^{5}\right\}\\ \left\{s_{2}^{5},s_{3}^{5},s_{4}^{5}\right\} & \left\{s_{2}^{5},s_{3}^{5},s_{4}^{5}\right\} & \left\{s_{1}^{5},s_{2}^{5}\right\} & \left\{s_{3}^{5}\right\}\\ \left\{s_{0}^{5},s_{1}^{5},s_{2}^{7}\right\} & \left\{s_{2}^{5},s_{3}^{5},s_{4}^{5}\right\} & \left\{s_{2}^{5}\right\} & \left\{s_{2}^{5}\right\} \end{array}\right]$
$H^{13}=\left[\begin{array}{cccc} \left\{s_{1}^{3},s_{2}^{3}\right\} & \left\{s_{0}^{3},s_{1}^{3},s_{2}^{3}\right\} & \left\{s_{1}^{3},s_{2}^{3}\right\} & \left\{s_{1}^{3},s_{2}^{3}\right\}\\ \left\{s_{1}^{5},s_{2}^{5},s_{3}^{5}\right\} & \left\{s_{2}^{5}\right\} & \left\{s_{3}^{5}\right\} & \left\{s_{3}^{5},s_{4}^{5}\right\}\\ \left\{s_{3}^{7},s_{4}^{7},s_{5}^{7}\right\} & \left\{s_{3}^{7},s_{4}^{7},s_{5}^{7}\right\} & \left\{s_{3}^{7},s_{4}^{7}\right\} & \left\{s_{3}^{7},s_{4}^{7},s_{5}^{7},s_{6}^{7}\right\}\\ \left\{s_{3}^{5}\right\} & \left\{s_{2}^{5},s_{3}^{5},s_{4}^{5}\right\} & \left\{s_{1}^{5},s_{2}^{5},s_{3}^{5}\right\} & \left\{s_{1}^{5},s_{2}^{5},s_{3}^{5},s_{4}^{5}\right\} \end{array}\right]$	$H^{14}=\left[\begin{array}{cccc} \left\{s_{1}^{3}\right\} & \left\{s_{0}^{3},s_{1}^{3}\right\} & \left\{s_{1}^{3},s_{2}^{3}\right\} & \left\{s_{2}^{3}\right\}\\ \left\{s_{1}^{5}\right\} & \left\{s_{0}^{5},s_{1}^{5},s_{2}^{5}\right\} & \left\{s_{1}^{5},s_{2}^{5},s_{3}^{5}\right\} & \left\{s_{4}^{5}\right\}\\ \left\{s_{2}^{7}\right\} & \left\{s_{2}^{7},s_{3}^{7}\right\} & \left\{s_{3}^{7},s_{4}^{7},s_{5}^{7}\right\} & \left\{s_{3}^{7},s_{4}^{7},s_{5}^{7},s_{6}^{7}\right\}\\ \left\{s_{2}^{7},s_{3}^{7},s_{4}^{7}\right\} & \left\{s_{3}^{7},s_{4}^{7}\right\} & \left\{s_{3}^{7},s_{4}^{7},s_{5}^{7}\right\} & \left\{s_{4}^{7},s_{5}^{7},s_{6}^{7}\right\} \end{array}\right]$
$H^{15}=\left\{\begin{array}{cccc} \left\{s_{1}^{3},s_{2}^{3}\right\} & \left\{s_{0}^{3},s_{1}^{3},s_{2}^{3}\right\} & \left\{s_{1}^{3},s_{2}^{3}\right\} & \left\{s_{1}^{3},s_{2}^{3}\right\}\\ \left\{s_{1}^{5},s_{2}^{5},s_{3}^{5}\right\} & \left\{s_{1}^{5},s_{2}^{5}\right\} & \left\{s_{2}^{5},s_{3}^{5}\right\} & \left\{s_{2}^{5},s_{3}^{5}\right\}\\ \left\{s_{1}^{7},s_{2}^{7}\right\} & \left\{s_{2}^{7},s_{3}^{7},s_{4}^{7},s_{5}^{7}\right\} & \left\{s_{3}^{7},s_{4}^{7},s_{5}^{7},s_{6}^{7}\right\} & \left\{s_{1}^{7},s_{2}^{7},s_{3}^{7},s_{4}^{7}\right\}\\ \left\{s_{2}^{7},s_{3}^{7},s_{4}^{7}\right\} & \left\{s_{3}^{7},s_{4}^{7},s_{5}^{7},s_{6}^{7}\right\} & \left\{s_{5}^{7},s_{6}^{7}\right\} & \left\{s_{4}^{7}\right\} \end{array}\right\}$	$H^{16}=\left[\begin{array}{cccc} \left\{s_{2}^{7},s_{3}^{7},s_{4}^{7}\right\} & \left\{s_{2}^{7},s_{3}^{7},s_{4}^{7},s_{5}^{7}\right\} & \left\{s_{2}^{7},s_{3}^{7},s_{4}^{7}\right\} & \left\{s_{4}^{7},s_{5}^{7},s_{6}^{7}\right\}\\ \left\{s_{1}^{7},s_{2}^{7},s_{3}^{7}\right\} & \left\{s_{2}^{7},s_{3}^{7}\right\} & \left\{s_{4}^{7}\right\} & \left\{s_{5}^{7},s_{6}^{7}\right\}\\ \left\{s_{2}^{7}\right\} & \left\{s_{2}^{7},s_{3}^{7},s_{4}^{7},s_{5}^{7}\right\} & \left\{s_{3}^{7},s_{4}^{7},s_{5}^{7}\right\} & \left\{s_{3}^{7},s_{4}^{7}\right\}\\ \left\{s_{3}^{7}\right\} & \left\{s_{3}^{7},s_{4}^{7},s_{5}^{7},s_{6}^{7}\right\} & \left\{s_{5}^{7},s_{6}^{7}\right\} & \left\{s_{5}^{7}\right\} \end{array}\right]$
$H^{17}=\left\{\begin{array}{cccc} \left\{s_{1}^{7},s_{2}^{7},s_{3}^{7},s_{4}^{7}\right\} & \left\{s_{3}^{7},s_{4}^{7},s_{5}^{7}\right\} & \left\{s_{3}^{7},s_{4}^{7}\right\} & \left\{s_{4}^{7},s_{5}^{7},s_{6}^{7}\right\}\\ \left\{s_{1}^{7},s_{2}^{7}\right\} & \left\{s_{3}^{7}\right\} & \left\{s_{4}^{7}\right\} & \left\{s_{4}^{7},s_{5}^{7},s_{6}^{7}\right\}\\ \left\{s_{2}^{7},s_{3}^{7}\right\} & \left\{s_{1}^{7},s_{2}^{7},s_{3}^{7},s_{4}^{7},s_{5}^{7}\right\} & \left\{s_{4}^{7},s_{5}^{7}\right\} & \left\{s_{3}^{7},s_{4}^{7},s_{5}^{7},s_{6}^{7}\right\}\\ \left\{s_{2}^{7},s_{3}^{7}\right\} & \left\{s_{4}^{7},s_{5}^{7},s_{6}^{7}\right\} & \left\{s_{5}^{7},s_{6}^{7}\right\} & \left\{s_{5}^{7},s_{6}^{7}\right\} \end{array}\right\}$	$H^{18}=\left[\begin{array}{cccc} \left\{s_{1}^{5},s_{2}^{5}\right\} & \left\{s_{2}^{5},s_{3}^{5}\right\} & \left\{s_{2}^{5},s_{3}^{5}\right\} & \left\{s_{3}^{5},s_{4}^{5}\right\}\\ \left\{s_{1}^{5}\right\} & \left\{s_{1}^{5},s_{2}^{5}\right\} & \left\{s_{1}^{5},s_{2}^{5}\right\} & \left\{s_{3}^{5}\right\}\\ \left\{s_{1}^{5}\right\} & \left\{s_{0}^{5},s_{1}^{5}\right\} & \left\{s_{2}^{5},s_{3}^{5},s_{4}^{5}\right\} & \left\{s_{2}^{5},s_{3}^{5},s_{4}^{5}\right\}\\ \left\{s_{1}^{5}\right\} & \left\{s_{0}^{5},s_{1}^{5},s_{2}^{5},s_{3}^{5}\right\} & \left\{s_{2}^{5},s_{3}^{5}\right\} & \left\{s_{4}^{5}\right\} \end{array}\right]$
$H^{19}=\left[\begin{array}{cccc} \left\{s_{0}^{3},s_{1}^{3}\right\} & \left\{s_{1}^{3}\right\} & \left\{s_{1}^{3},s_{2}^{3}\right\} & \left\{s_{1}^{3},s_{2}^{3}\right\}\\ \left\{s_{1}^{3}\right\} & \left\{s_{1}^{3},s_{2}^{3}\right\} & \left\{s_{1}^{3},s_{2}^{3}\right\} & \left\{s_{1}^{3}\right\}\\ \left\{s_{1}^{3}\right\} & \left\{s_{1}^{3},s_{2}^{3}\right\} & \left\{s_{1}^{3}\right\} & \left\{s_{1}^{3}\right\}\\ \left\{s_{0}^{3},s_{1}^{3}\right\} & \left\{s_{1}^{3}\right\} & \left\{s_{2}^{3}\right\} & \left\{s_{1}^{3},s_{2}^{3}\right\} \end{array}\right]$	$H^{20}=\left[\begin{array}{cccc} \left\{s_{1}^{5},s_{2}^{5},s_{3}^{5}\right\} & \left\{s_{2}^{5},s_{3}^{5}\right\} & \left\{s_{3}^{5},s_{4}^{5}\right\} & \left\{s_{2}^{5},s_{3}^{5},s_{4}^{5}\right\}\\ \left\{s_{1}^{5},s_{2}^{5}\right\} & \left\{s_{1}^{5},s_{2}^{5}\right\} & \left\{s_{2}^{5},s_{3}^{5}\right\} & \left\{s_{4}^{5}\right\}\\ \left\{s_{2}^{7}\right\} & \left\{s_{2}^{7},s_{3}^{7},s_{4}^{7}\right\} & \left\{s_{3}^{7},s_{4}^{7},s_{5}^{7}\right\} & \left\{s_{3}^{7},s_{4}^{7},s_{5}^{7},s_{6}^{7}\right\}\\ \left\{s_{2}^{7},s_{3}^{7}\right\} & \left\{s_{4}^{7},s_{5}^{7},s_{6}^{7}\right\} & \left\{s_{5}^{7},s_{6}^{7}\right\} & \left\{s_{4}^{7},s_{5}^{7},s_{6}^{7}\right\} \end{array}\right]$
