# Correction: Machine-assisted adaptive feedback consensus method for large-scale group decision making based on reinforcement learning

**DOI:** 10.3389/fdata.2026.2014647

**Published:** 2026-10-26

**Authors:** Hongyu Yu, Xuanhua Xu, Weiwei Zhang

**Affiliations:** 1 School of Advanced Interdisciplinary Studies, Hunan University of Technology and Business, Changsha, China; 2 School of Business, Central South University, Changsha, China

**Keywords:** adaptive feedback mechanism, consensus reaching, human-machine collaboration, large-scale group decision-making, reinforcement learning

In the published article, the funder Major Program of the National Natural Science Foundation of China, 72293574 and Key Program of Xiangjiang Laboratory, 25XJ02007 to Xuanhua Xu was erroneously omitted.

In addition, the funder Outstanding Youth Project of the Education Department of Hunan Province, 24B0566 to Weiwei Zhang was erroneously omitted.

The correct **Funding** statement appears below:

The author(s) declared that financial support was received for this work and/or its publication. This research was based upon work supported by the Major Program of the National Natural Science Foundation of China (grant number 72293574), the Key Program of Xiangjiang Laboratory (grant number 25XJ02007) to Xuanhua Xu, and the Outstanding Youth Project of the Education Department of Hunan Province (grant number 24B0566) to Weiwei Zhang.

The original version of this article has been updated.

